# The Association Between Hospital Financial Performance and the Quality of Care – A Scoping Literature Review

**DOI:** 10.34172/ijhpm.2022.6957

**Published:** 2022-08-16

**Authors:** Katarzyna Dubas-Jakóbczyk, Ewa Kocot, Marzena Tambor, Przemysław Szetela, Olga Kostrzewska, Richard B. Siegrist Jr, Wilm Quentin

**Affiliations:** ^1^Health Economics and Social Security Department, Institute of Public Health, Faculty of Health Sciences, Jagiellonian University Medical College, Krakow, Poland.; ^2^Institute of Public Health, Faculty of Health Sciences, Jagiellonian University Medical College, Krakow, Poland.; ^3^Harvard T.H. Chan School of Public Health, Boston, MA, USA.; ^4^Department of Health Care Management, Technische Universität Berlin, Berlin, Germany.; ^5^European Observatory on Health Systems and Policies, WHO European Centre for Health Policy Eurostation (Office 07C020), Brussels, Belgium.

**Keywords:** Hospital, Financial Performance, Profit, Quality of Care, Indicator

## Abstract

**Background:** Improving the quality of hospital care is an important policy objective. Hospitals operate under pressure to contain costs and might face challenges related to financial deficits. The objective of this paper was to identify and map the available evidence on the association between hospital financial performance (FP) and quality of care (Q).

**Methods:** A scoping review was performed. Searches were conducted in 7 databases: Medline via PubMed, EMBASE, Web of Science, Scopus, EconLit, ABI/INFORM, and Business Source Complete. The search strategy combined multiple terms from 3 topics: hospital AND FP AND Q. The collected data were analysed using both quantitative and qualitative methods.

**Results:** 10 503 records were screened and 151 full text papers analysed. A total of 69 papers were included (60 empirical, 2 theoretical, 5 literature reviews, and 2 dissertations). The majority of identified studies were published within the last decade (2010-2021). Most empirical studies had been conducted in the United States (55/60), used cross-sectional approaches (32/60) and applied diverse regression models with FP measures as dependent variables, thus measuring the impact of Q on hospitals FP (34/60). The comparability of the studies’ results is limited due to differences in applied methods and settings. Yet, the general overview shows that in almost half of the cases the association between hospital FP and Q was positive, while no study showed a clear negative association.

**Conclusion:** This scoping review provides an overview of the available literature on the association between hospital FP and Q. The results highlight numerous research gaps: (1) systematic reviews and meta-analyses of existing studies with similar measures of FP and Q are unavailable, (2) further methodological/conceptual work is needed on the metrics measuring hospital FP and Q, and (3) more empirical studies should analyse the association between FP and Q in non-US healthcare settings.

## Background

 Improving the quality of hospital care has been an important policy objective for more than three decades.^1-3^ Numerous organizations have developed guidelines on strategies aimed at quality improvement in hospital settings,^4,5^ while the scope of the empirical evidence on the effectiveness of different approaches is also growing.^2,3,6^ At the same time, hospitals around the world are operating under pressure to contain costs^7,8^ and might face challenges related to financial deficits.^9-11^ The potential trade-off between costs and quality in healthcare has been broadly discussed in the literature^1,12,13^ with a recent systematic review of empirical studies on the relationship between hospital costs/prices and the quality of care (Q)^14^ showing mixed results.

**Figure 1:**
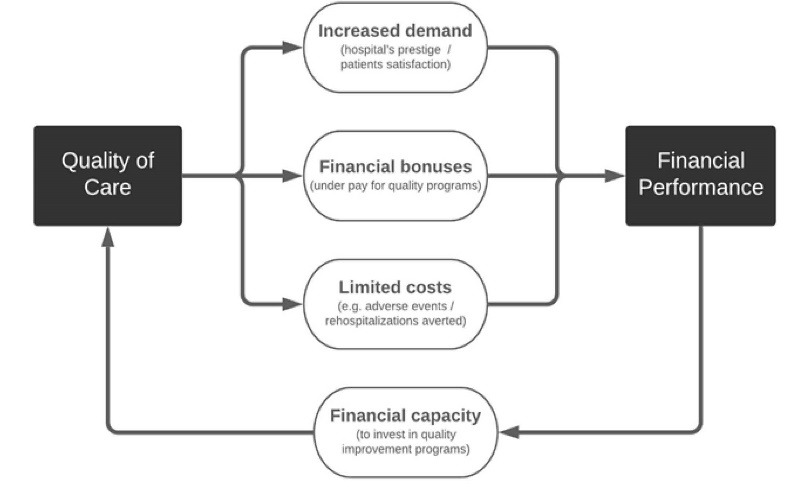
General Overview of the Association Between Hospital Financial Performance and Quality of Care. Source: authors’ own work.

 In general, the literature suggests that the association between hospital financial performance (FP) and the Q can go in two directions.^15,16^ On the one hand, high Q might lead to better financial outcomes. This can be the case if Q is an important determinant of patient choice of provider,^17,18^ thus increasing demand and hospital revenues. For example, a study from the United States showed that improvement of a hospital’s ranking in the publicly reported quality metrics was associated with a 5% increase in the number of patients.^19^ In addition, pay for quality or pay for performance programmes may provide financial bonuses to hospitals meeting pre-defined quality standards.^6^ Finally, Q can also improve hospital FP by generating savings and avoiding waste, eg, by avoiding the costs of adverse events or rehospitalizations. On the other hand, hospitals that are more financially stable (eg, generating profits) will likely have greater capacity to invest in quality improvement. This may involve paying higher wages and employing better specialists as well as investing in modern information technology solutions which support quality improvement programmes (Figure 1). Nevertheless, the scope and the underlying mechanisms of these associations have not yet been thoroughly analysed.

 Two previous literature reviews on the association between hospital FP and Q exist. However, one included only studies from the United States,^15^ while the other was conducted more than a decade ago.^16^ Also, both reviews included studies where FP was measured by proxy data, eg, solely costs or revenues, and not the actual FP ratio (eg, the relation of revenues to costs). There are also several literature reviews/studies focusing on or including the evaluation of pay for quality/performance programmes in hospital settings.^6,21,21^ Yet, in the case of those, the authors were mainly interested in the impact of participation in the incentive program on Q (eg, patient outcomes), while the overall hospital financial standing was not analysed.

 The general objective of this scoping review was to identify and map the available evidence on the association between hospital FP and Q. As both the hospitals’ ‘FP’ and ‘Q’ constitute complex and multidimensional concepts, we aimed to classify the available literature and provide a broad overview of the topic. We have not applied a publication date limit and have included both quantitative and qualitative empirical studies as well as theoretical papers and grey literature. In addition, this scoping review aimed to identify the gaps in the literature and to define research questions for a future systematic review.

## Methods

 The review follows the six-stage methodological framework outlined by Arksey and O’Malley^22^ and further developed by Levac et al.^23^ For the reporting we have utilized the PRISMA extension for Scoping Reviews (PRISMA-ScR) checklist^24^ (Supplementary file 1). The final searches were conducted in August 2021. This project has been registered through the Open Science Framework^25^ and the review protocol has been published in a peer-reviewed journal.^26^

###  Defining the Research Questions

 The following specific research questions (RQ) were formulated:

- RQ1 – What types of studies/papers were conducted/published?
- RQ2 – What type of conceptual/theoretical framework was applied?
- RQ3 – What type of association was being assessed?
- RQ4 – How was the FP defined and measured?
- RQ5 – How was the Q defined and measured?
- RQ6 – What association was identified?
- RG7 – What limitations were stated?

###  Identifying Relevant Literature

 Searches were conducted in seven electronic databases: (1) Medline via PubMed, (2) EMBASE via OVID, (3) the Web of Science Core Collection, (4) Scopus, (5) EconLit, (6) ABI/INFORM, and (7) Business Source Complete. The search strategy combined terms from three topics: (1) hospital AND (2) financial performance AND (3) quality of care (Table 1). Terms were searched as keywords in the title and/or abstract without a publication date limit.

**Table 1:** Search Terms for the Databases

**Topic**	**Search Terms**
Hospital	hospital* OR inpatient*
Financial performance	financial performance OR financial standing OR financial situation OR financial indicator* OR financial condition* OR financial failure OR financial distress OR financial measure* OR financial parameter* OR profit* OR operating margin* OR cash flow OR debt* OR liquidity OR asset turnover
Quality of care	quality OR staff* OR technology OR health outcome* OR patient* safety OR patient* satisfaction OR readmission* OR adverse event* OR complication*

 The reference lists of relevant paperswere visually scanned with the aim of identifying further studies of interest. Also, grey literature was searched by screening the websites of 21 international and national organizations dealing with hospital performance and/or Q. Supplementary file 2 presents a list of websites screened as well as the operationalisation of the search strategy for the different databases.

###  Study Selection 

 The Mendeley reference manager was used for the record selection process. The selection consisted of two stages: (1) screening a title and abstract and (2) a full-text review. For the first level of screening two researchers (authors of this paper) screened a random 10% sample of records, and compared and discussed their results until consensus was reached. The agreement between them was sufficiently high (92% raw agreement), thus the remaining records were screened by one researcher. The full text articles were assessed independently by two researchers according to the pre-defined inclusion and exclusion criteria. The inclusion and exclusion criteria were as follows:

1. Inclusion: both FP and Q are defined and measured; exclusion: only one dimension is measured.
2. Inclusion: FP is measured by an FP ratio, ie, a ratio of revenues to costs; exclusion: only proxy data is used, eg, only costs or revenues measures.
3. Inclusion: the focus is on the hospital setting; exclusion: studies conducted in nursing homes.
4. Inclusion: the association between FP and Q is assessed; exclusion: there is no analysis of association between FP and Q.
5. Inclusion: the publication is a peer-reviewed empirical study or theoretical paper, technical report, book/chapter, thesis; exclusion: conference abstracts.
6. Inclusion: the full text is available in English; exclusion: only the abstract is available in English.

Any discrepancies between the two researchers were addressed by consulting the third researcher who took a final decision on paper inclusion.

###  Data Extraction

 Two data extraction and coding templates were developed by the research team: one for empirical studies and another one for other types of studies. Supplementary file 3 presents the data extraction templates. Each section of the templates is related to a specific research question with specific codes assigned for further analysis (where appropriate). In studies with multiple objectives, only the data related to the association between FP and Q were extracted. Data extraction was an iterative process, with the data from a random sample of 10% of the studies extracted by two researchers (authors of this study) independently. Results were then compared and any discrepancies were discussed to ensure consistency. Agreement between the two researchers was sufficiently high (88% raw agreement), thus the data of the remaining studies was extracted by one researcher only.

###  Collating, Summarizing and Reporting the Results 

 The collected data were analysed using both quantitative and qualitative (thematic analysis) methods. We classified the empirical studies based on the metrics used to operationalize the concepts of FP and Q: single indicators, multiple indicators and/or complex (composite) measures. In addition, studies were categorized based on existing concepts, ie, the type of ratio analysis used to measure FP (eg, profitability ratio or liquidity ratio, see Gapenski and Pink^27^), and Donabedian’s triad of structure, process, and outcome measures for Q.^28^ For Q measures, we also classified studies based on the main dimension of quality assessed (ie, adverse events, readmission, patient satisfaction, etc).

 Data on the identified associations was extracted by focusing on the results of the statistical analysis and the significance level, followed by coding the overall association between FP and Q as: P – positive, N – negative, L – lack, mixed. For example, in the case of studies where Q was measured by readmission ratios, and the statistical analysis showed a negative, statistically significant correlation – ie, with a decrease in the number of readmissions, the hospital profits increased – the overall association between Q and hospital FP was coded as positive – ie, an improvement in quality was correlated with an improvement in FP.

###  Consultation Process and Engagement of Knowledge Users

 The preliminary findings were shared with the relevant stakeholders during a scientific seminar held by the leading author’s university department and presented at an international (European Public Health) conference.^29^

## Results

###  Search Results

**Figure 2:**
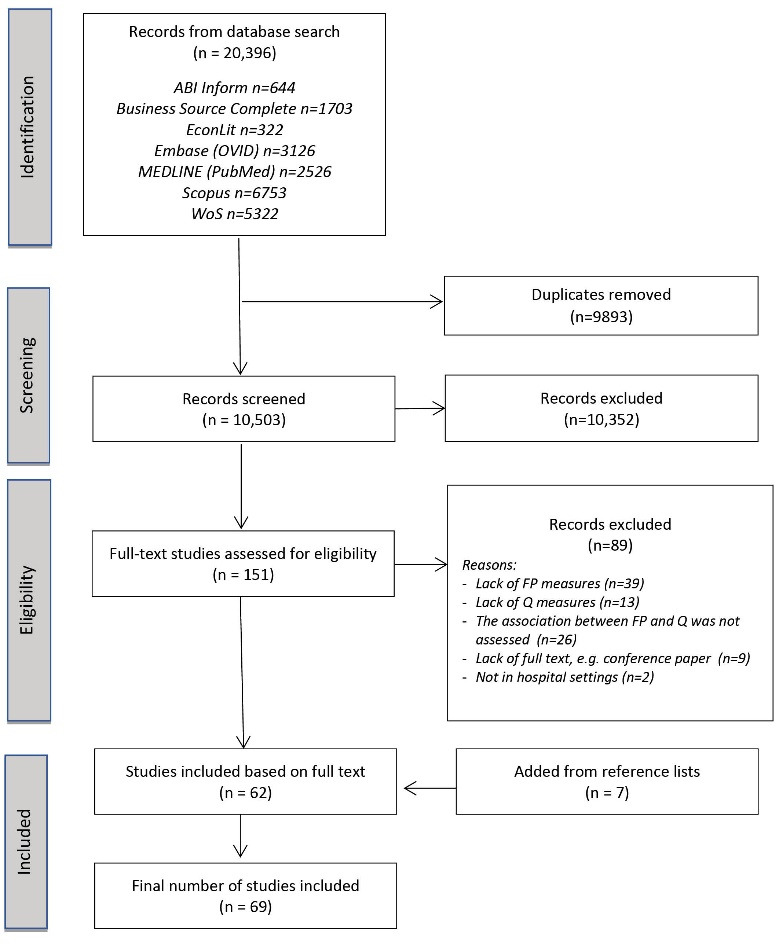
PRISMA Flow Diagram. Abbreviations: PRISMA, Preferred Reporting Items for Systematic Reviews and Meta-Analyses; FP, financial performance; Q, quality of care.

 The search of seven databases identified 20,396 relevant citations. Supplementary file 2 presents results for each database. After removing duplicates, 10 503 records were screened. Based on titles and abstracts, 151 full text papers were obtained for further analysis, of which 89 studies were excluded for not meeting the eligibility criteria. The most common reason for exclusion was the lack of FP measures, eg, only costs or expenditures were presented. After full-text analysis, 62 papers were classified as meeting the eligibility criteria. Screening their reference lists resulted in the inclusion of an additional 7 papers. Therefore, a total number of 69 papers were included^15,16,30-97^ as presented in the PRISMA flow diagram (Figure 2). Supplementary file 4 presents the list of included studies by year and type of publication, with full reference data.

###  Types of Studies

**Figure 3:**
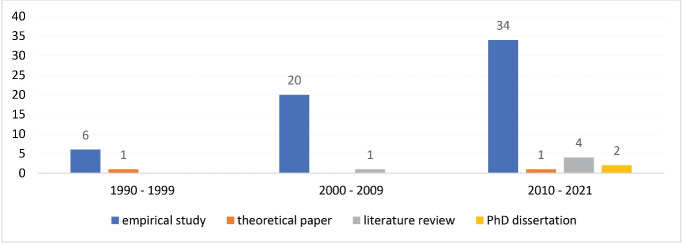
Number and Type of Included Studies Per Publication Period.

 Among the 69 included papers there are 60 empirical studies,^37-96^ two theoretical papers,^30,31^ five literature reviews,^15,16,32-34^ and two PhD dissertations.^35,36^ The majority of studies (59%, 41/69) were published during the last decade (between 2010 and 2021) (Figure 3). Among the 60 empirical studies, a vast majority (92%, 55/60) were focused on the US market. Only five of the identified empirical studies were conducted in other countries: two in the United Kingdom,^78,93^ one each in Japan,^56^ Belgium^96^ and Austria.^91^ Of the five literature reviews two were focused on the association between FP and Q,^15,16^ yet covered a rather broad FP definition (eg, including studies where only costs and/or revenue indicators were used). One review focused on the association between the hospital’s clinical technology and financial outcomes^33^ while the remaining two provided a rather broad overview of studies on different organizational (including Q) and environmental factors affecting hospital FP.^32,34^ The two theoretical papers^30,31^ provided descriptions of multidimensional conceptual frameworks linking elements of quality and financial measures. Finally, the two PhD dissertations were also focused on the US hospital market and included regional, empirical analyses on the association between clinical quality^35^ or patient satisfaction^36^ and FP (Supplementary file 5).

###  Conceptual Framework Used

 In about half of the empirical papers (29/60) the authors described a conceptual/theoretical framework used for analysing the association between FP and Q (Supplementary file 6). Often the authors made direct reference to some previously published theory. For example: the economic theory of hospital behaviour based on Newhouse^97^ and/or Hoerger^98^ was referred to in four studies^79,81,82,86^ while the resource-based theory by Barney^99^ or the resource dependence theory by Pfeffer and Salancik^100^ were referred to in four^47,55,77,90^ and three^67,73,74^ studies respectively. Other studies developed their own frameworks, which postulated how different factors embedded in a hospitals’ internal (including Q) and/or external environment can affect its FP.^49,57-59,84,86^

###  Empirical Studies Overview

Table 2 presents a general overview of the 60 empirical studies, ordered according to the type of association assessed.

**Table 2:** Empirical Studies Overview

**Study**			**Methods**			**FP**			**Quality**			**Outcome**	**Limitations** ^g^
**Ref.**	**First Author/s and Publication Year**	**Country**	**Study Design** ^a^	**Hospital Sample** ^b^	Type/Direction of Association Being Assessed*	**Level** ^c^	**Category** ^d^	**Measures** ^e^	**Area**	**Category** ^f^	**Measures** ^e^	Overall Assessment of Association**	
37	Alexander et al/2006	US	LT	N	Q→FP	H	O	S	Mix	S, P	M	Mixed	n/s
38	Ammori et al/2007	US	CS	CS	Q→FP	H	P	S	Adverse events	O	S	P	D, M
39	Ammori et al/2008	US	CS	CS	Q→FP	H	P	S	Adverse events	O	M	L	M
40	Asagbra et al/2019	US	LT	N	Q→FP	H	P, O	M	HIT**	S	C	P	D
41	Beauvais et al/2019	US	CS	N	Q→FP	H	P	M	Mix	S, P, O	C	P	D, M
42	Brooks et al/2021	US	CS	R	Q→FP	H	P	S	Staff	S	S	L	D, M
43	Clement et al/2014	US	LT	CS	Q→FP	P	P	M	Readmissions	P	S	Mixed	D
44	Dimick et al/2006	US	CS	CS	Q→FP	P	P	M	Adverse events	O	C	P	D, M
45	Eappen et al/2013	US	CS	R	Q→FP	P	P	S	Adverse events	O	M	Mixed	D, M
46	Englesbe et al/2006	US	CS	CS	Q→FP	P	P	S	Adverse events	O	S	P	D, M
47	Everhart et al/2013	US	CS	R	Q→FP	H	P	S	Staff	S	S	Mixed	D, M
48	Flynn et al/2014	US	CS	CS	Q→FP	P	P	S	Adverse events	O	S	P	D
49	Harkey and Vraciu/1992	US	CS	R	Q→FP	H	P	S	Mix	P	C	P	n/s
50	Healy et al/2016	US	LT	CS	Q→FP	P	P	S	Adverse events	O	C	P	D
51	Hegji and Self/2007	US	CS	R	Q→FP	P	P	M	Process of care	S, P	M	Mixed	D
52	Hegji and Self/2009	US	CS	N	Q→FP	P	P	S	Process of care	P	M	Mixed	n/s
53	Hegji/2006	US	CS	R	Q→FP	H	P	S	Process of care	P	M	L	D
54	Ho et al/2010	US	CS	CS	Q→FP	P	P	S	Adverse events	O	S	P	D, M
55	Irwin et al/1998	US	LT	R	Q→FP	H	P	M	HIT	S	C	P	D
56	Kodera and Yoneda/2015	Japan	CS	R	Q→FP	H	P	S	Accreditation	S, P	M	L	D
57	Li and Collier/2000	US	CS	N	Q→FP	H	P, O	M/C	Mix	P, O	M/C	Mixed	D
58	Lim et al/2018	US	CS	R	Q→FP	H	P	M	Patient satisfaction	P, O	M	P	D
59	Maiga and Jacobs/2009	US	CS	N	Q→FP	H	P	M	Patient satisfaction	O	S	P	D, M
60	McCue et al/2003	US	LT	R	Q→FP	H	P	S	Mix	S, O	M	L	D, M
61	Nelson et al/1992	US	CS	R	Q→FP	H	P, O	M	Patient satisfaction	P, O	M	P	D, M
62	Nevola et al/2016	US	LT	R	Q→FP	H	P, O	M	Patient satisfaction	P,O	M	L	D
63	Parente et al/2001	US	LT	N	Q→FP	H	P	M	HIT	S	S	P	D
64	Reiter et al/2012	US	LT	N	Q→FP	H	P	S	Staff	S	S	Mixed	D
65	Richter and Muhlestein/2017	US	LT	N	Q→FP	H	P	M	Patient satisfaction	P, O	M	P	M
66	Self et al/2010	US	CS	R	Q→FP	P	P	M	Process of care	P	M	Mixed	D
67	Upadhyay et al/2019	US	LT	R	Q→FP	H	P	S	Readmissions	P	S	Mixed	D, M
68	Wang et al/2018	US	LT	N	Q→FP	H	O	S	HIT	S	S	P	D
69	Wright et al/2016	US	CS	N	Q→FP	H	P	S	HIT	P	S	L	D
70	Zhao et al/2019	US	CS	N	Q→FP	H/P	P, O	M	HIT	S	S	P	D, M
71	Dong/2015	US	LT	N	FP→Q	H	P, O	M	Mix	P	C	P	D
72	Encinosa and Bernard/2005	US	LT	R	FP→Q	H	P	S	Adverse events	O	M	Mixed	D
73	Ginn et al/2011	US	CS	N	FP→Q	H	P, O	M	HIT	S	C	Mixed	D, M
74	Kazley and Ozcan/2007	US	CS	N	FP→Q	H	P	S	HIT	S	S	L	D
75	Lindrooth et al/2013	US	LT	N	FP→Q	P	P	M/C	Mortality	O	S	P	M
76	Ly et al/2011	US	CS	N	FP→Q	H	P	S	Mix	P, O	M	Mixed	M
77	Menachemi et al/2006	US	CS	R	FP→Q	H	P, O	M	HIT	S	M/C	P	D, M
78	Nagendran et al/2019	UK	LT	R	FP→Q	H	P	S	Mix	P, O	M	Mixed	D, M
79	Navathe et al/2012	US	LT	N	FP→Q	P	P	S	Readmissions	P	S	L	M
80	Shen and Ginn/2012	US	LT	N	FP→Q	H	P, O	M	HIT	S	S/C	Mixed	D, M
81	Zhao et al/2008	US	LT	N	FP→Q	H	P, O	M	Staff	S	M	Mixed	D
82	Bazzoli et al/2007	US	LT	N	Both	H/P	P	M	Accreditation	S, P	M	P	D
83	Nguyen et al/2016	US	LT	R	Both	H	P	M	Readmissions	P, O	M	Mixed	D, M
84	Turner et al/2015	US	LT	R	Both	H	P, O	M	Mix	P, O	M/C	L	D
85	Akinleye et al/2019	US	CS	R	Lack of direction	H	P, O	C	Mix	P, O	C	Mixed	n/s
86	Bazzoli et al/2008	US	LT	R	Lack of direction	H	P	M	Adverse events	O	M	Mixed	D
87	Cleverley et al/1992	US	CS	N	Lack of direction	H	P	M	Mortality	O	S	P	D
88	Collum et al/2016	US	LT	N	Lack of direction	H	P	M	HIT	S	C	L	D
89	Cowan et al/2006	US	LT	CS	Lack of direction	P	P	S	Staff	S, P	C	P	n/s
90	Crowe et al/2017	US	CS	N	Lack of direction	H	P, O	M	Staff	S, P	C/M	P	D, M
91	Dauser et al/2021	Austria	CS	CS	Lack of direction	P	P	S	HIT	P	S	P	M
92	Hsia and Ahern 1992	US	CS	R	Lack of direction	P	P	S	Mix	P	M	P	n/s
93	Jenks et al/2014	UK	LT	CS	Lack of direction	P	P	S	Adverse events	O	S	Mixed	D
94	Karim et al/2018	US	LT	N	Lack of direction	H	P, O	M	Staff	P	C	P	D
95	Langland-Orban et al/1996	US	LT	R	Lack of direction	H	P	M	Accreditation	S, P	M	P	n/s
96	Pirson et al/2008	Belgium	CS	R	Lack of direction	P	P	S	Adverse events	O	S	P	D, M

Abbreviations: FP, financial performance; Q, quality of care; HIT, health information technology. *Direction of association between FP and Q is based on the dependent/independent variable in the regression model; **The overall assessment of the association between FP and Q (P – positive, N – negative, L – lack) is based on the results of statistical analyses. ^a^ CS, cross-sectional; LT, longitudinal. ^b^ N, national; R, regional; CS, case study. ^c^ H, hospital; P, patient, procedure. ^d^ P, profitability; O, other. ^e^ S, single; M, multiple; C, composite. ^f^ S, structure; P, process; O, outcome. ^g^ n/s, none stated; D, related to data; M, related to methods.

####  Methods Applied

 Most were observational studies, using either a cross-sectional (32) or a longitudinal (28) design. In the majority of cases, diverse forms of public registries/portals were used as data sources. Researchers utilized both national (25) and regional (24) hospital data samples. Some conducted case studies based on data from only one hospital (11). Most studies applied diverse types of regression analyses to analyse data. In 34 studies FP measures were used as dependent variables, thus the impact of Q on FP was assessed. In 11 studies the Q measures were dependent variables (impact of FP on Q), while in three cases researchers developed multiple models and both directions of association were assessed. In the remaining 12 studies, a simple correlation and/or group comparison were applied (thus the direction of the association was not defined). The most often used control variables in the regression models were hospital characteristics (eg, ownership, size, payer mix) and/or patient characteristics.

####  Financial Performance Dimensions and Measures 

 In the majority (41) of the empirical studies, the FP was measured at the hospital level. In 17 studies, indicators of profitability per patient/procedure were applied, while in two studies, multiple indicators were used – at both the hospital and the patient level. Researchers used both multiple (minimum two) and single indicators to measure FP, while in the case of three studies, only a composite measure was developed and applied. In general, regardless of the level (hospital vs. patient/procedure) and number of indicators used, diverse profitability indicators were dominant measures of FP (used in 58 out of 60 studies, including 14 studies where profitability indicators were used together with other FP measures, eg, return on assets).

####  Quality Dimensions and Measures 

 In terms of quality measures, analysis of the empirical studies showed much more diversity. The studies focused on the following quality areas/themes: existence and/or use of technological innovations, mainly health information technology (HIT) (most commonly level of a specified HIT adoption, including eg, systems for supporting clinical decisions or patients engagement) (12); adverse events (12); measures related to staff qualifications and/or size (7); patient satisfaction (5); readmissions (4); specific measures of process of care in predefined conditions (4); accreditation (3); mortality (2). In the case of the remaining 11 studies, the quality measures covered mixed areas/themes and applied multiple or composite indicators. The latter included, eg, studies where a composite quality metric was developed for the purposes of the study, including several process and outcome of care indicators^85^ as well as studies where existing composite measures were applied (eg, Leapfrog Hospital Safety Score comprising 28 unique factors^41^). In general, many studies (39/60) included multiple and/or composite indicators to analyse different aspects of Q. Half of the studies (30/60) included one or several process indicators, eg, compliance with a standard/recommended set of procedures for specific conditions, often in combination with indicators of outcomes (11) or structures (8). Almost as many studies (27/60) included one or several outcome indicators, often assessing the prevalence of adverse events (12) or patient satisfaction (5). Many studies also included structural indicators (23/60), often relating to the existence of HIT in hospitals (10) or the availability of certain staff (5).

####  Identified Associations

Table 3 shows that almost half of the studies (29/60) found a significant positive association between hospital FP and quality (ie, better quality was associated with better FP or vice versa). Positive associations between Q and FP were found for all quality areas except readmissions and processes of care, where results were mixed or not significant. Of the 34 studies which assessed the effect of Q on FP (FP as a dependent variable) 17 found a significant positive association. Of the 11 studies analysing the effect of FP on Q (quality measures as dependent variables) three studies found a significant positive association while six found mixed and two no significant results. In many studies (20/60) the results were mixed, eg, positive for some of the metrics/analyses and not significant for others. No study found a clear negative association between Q and FP. Negative associations were found only in studies using multiple models, with some showing positive and others negative associations, which were classified here as providing mixed results. For example, in one study on adverse events, the occurrence of surgical complications was associated with a higher hospital contribution margin per patient, yet the results varied significantly depending on the payer mix.^45^

**Table 3:** Overview of Identified Associations Per Type of Association and Quality Area

Type of Association*/Q Area	Overall Assessment of Association Between FP and Q, Per Number of Studies**				
	**Q→FP**	**FP→Q**	**Both**	**Lack of Direction**	**Sum**
HIT	P – 5 L – 1	P – 1 Mixed – 2 L – 1	-	P – 1 L – 1	P – 7 Mixed – 2 L – 3
Adverse events	P – 6 L – 1 Mixed – 1	Mixed – 1	-	P – 1 Mixed – 2	P – 7 Mixed – 4 L – 1
Staff	Mixed – 2 L – 1	Mixed – 1	-	P – 3	P – 3 Mixed – 3 L – 1
Patient satisfaction	P – 4 L – 1	-	-	-	P – 4 L – 1
Readmissions	Mixed – 2	L – 1	Mixed – 1	-	Mixed – 3 L – 1
Process of care	Mixed – 3 L – 1	-	-	-	Mixed – 3 L – 1
Accreditation	L – 1	-	P – 1	P – 1	P – 2 L – 1
Mortality	-	P – 1	-	P – 1	P – 2
Mix	P – 2 Mixed – 2 L – 1	P – 1 Mixed – 2	L – 1	P – 1 Mixed – 1	P – 4 Mixed – 5 L – 2
**Sum**	P – 17 Mixed – 10 L – 7	P – 3 Mixed – 6 L – 2	P – 1 Mixed – 1 L – 1	P – 8 Mixed – 3 L – 1	P – 29 Mixed – 20 L – 11

Abbreviations: FP, financial performance; Q, quality of care; HIT, health information technology. *Direction of association between FP and Q, based on dependent/independent variable in regression model; **The overall assessment of the association between FP and Q (P – positive, N – negative, L – lack), based on the results of statistical analyses.

####  Studies Limitations

 In the vast majority of the empirical studies (53 out of 60), the authors indicated important limitations. They were related to the data (27), the methods (6) or both data and methods (20). Data limitations focused mostly on data quality or relatively small sample sizes (often mentioned in studies with regional samples or individual hospital case studies) which might have limited the generalizability of results. Limitations related to the methods focused mainly on the observational character of the studies, which made it possible to identify association, but not causality. There were also limitations specific to a particular quality measure. For example, in six studies focused on adverse events^38,39,44-46,54^ the authors pointed out that they had not assessed the opportunity cost of adverse events, which can prevent potentially profitable patients from occupying hospital beds (thus impacting hospital FP).

## Discussion

 The relationship between hospital FP and Q has been debated for several decades and two previous reviews have summarized the available literature.^15,16^ However, our review is more recent and relies on a more precise definition of FP. We have excluded studies focusing on proxy measures of solely costs or solely revenues. Such studies are problematic because FP does not necessarily improve if an increase in revenues is offset by a simultaneous increase in costs. Consequently, an association between increasing revenues and improved quality does necessarily indicate an association between quality and FP. Analogically, when improvement in Q is accompanied by increasing cost, this does not have to lead to worsening financial standing if there is also adequate revenue growth. Thus in our review we have included studies with actual indicators of FP, eg, ratios showing the relation of revenues to costs, mostly diverse profitability measures. This review also has a broader geographic scope (includes studies from outside the United States). It provides a broad overview of the available literature, identifies groups of studies using similar methodological approaches, and summarizes the results for different groups of studies.

 We identified 69 publications on the association between hospital FP and Q, published between 1992 and 2021. Most empirical studies were from the United States (55/60), used a cross-sectional approach (32/60), and applied diverse regression models (48/60). The majority of studies evaluated the effect of FP on Q; others assessed the effect of Q on FP; while still others looked at both directions or applied simple correlation/group comparison analyses (the direction of association was not defined). Despite important limitations with regard to the comparability of studies’ results due to differences in data, methods, and settings, our overview shows that almost half of all studies found a significant positive association between hospital FP and quality, while no study found a clear negative association (Table 3).

 Previous studies have postulated that there is a trade-off between quality and costs.^12,13^ However, our results seem to suggest that a clear trade-off between quality and FP is rather unlikely. The existing evidence points in the direction of a positive relationship between quality and hospital FP. This means that, at least for certain conditions or in certain contexts, hospitals can simultaneously improve and/or maintain quality and their financial standing (measured mainly by profits). However, given that some of the studies included in the review used relatively simple quality metrics, eg, the existence and use of HIT or the level of inpatient mortality, it is possible that results would be different if more comprehensive quality metrics were used, and if quality was evaluated separately for different clinical conditions. Simple structural indicators, eg, existence of specified HIT or the number of nurses per patient, are problematic also because the link to actual patient care is rather indirect. Similarly, results might be different if FP was measured using more comprehensive indicators that better reflect the multidimensional nature of a hospital’s financial standing.^101^ For example the value of total profit in a given year, can be easily influenced by ad hoc financial transfers/accounting decision. Our results confirm that the association between hospital FP and the Q is complex and multidimensional. Existing theoretical frameworks that are often based on economic and/or institutional theories do not establish a direct link between quality and FP. However, they usually point to a diversity of factors, both internal and external, that might impact hospital performance in terms of both the Q and financial standing. Also, as with any other type of investments, the implementation of quality improvement programs, requires an adequate timeframe and financial resources (eg, start-up costs). In other words, the choice of both the variables and methods to assess the association matters. The variables relate to both, the metrics used to measure Q and FP as well as the control variables. In term of the methods, a longitudinal approach is better equipped to capture the association between the two dimensions (eg, the time needed to successfully implement a quality improvement program).

 The majority of the identified studies (both overall and empirical ones) were published within the last decade (2010-2021) which suggests a growing interest in this area of research. However, a vast majority of the empirical studies were conducted in the United States. This indicates both the significant importance and interest in the topic in that country, as well as the related issue of data availability. The hospital sector in the United States has developed as a historically commercial market with an abundance of literature/research focused on hospital financial distress and/or bankruptcy issues.^102-104^ The broad scope of the hospital financial data is available via diverse administrative registers, eg, the Healthcare Cost Report Information System operated by the Centers for Medicare and Medicaid Services^105^ or the database operated by the American Hospital Association.^106^ These types of registers constituted the most common source of financial data in the included studies with a regional or national scope. At the same time, the issues related to quality assurance and monitoring have been strongly embedded in the US system for many years now, with an expansion of hospital care quality data available via public reporting schemes, eg, the Hospital Compare portal at the national level^107^ or regional healthcare Q metrics databases, like the Statewide Planning and Research Cooperative System for the state of New York.^108^

 The identification of only a few studies on the association between FP and Q conducted in other countries might be partially related to the lack of data availability. For example, among the four empirical studies conducted in European countries: two were case-studies utilizing primary data from only one hospital^91,93^; one primarily comprised data from three hospitals,^96^ while one study only utilized the data available via regional administrative registers.^78^ In general, in European countries the systems for publicly reporting hospital quality metrics are much less developed than in the United States.^109^ Also, the issue of hospital’s FP might be perceived in Europe from a different perspective than in the United States. In many European countries hospitals exist in a traditionally public sector (public ownership and financing). In many countries, the issue of hospital debts constitutes a permanent concern.^10^ Yet, to the authors best knowledge there seem to be no literature available on the issues related to measuring and monitoring hospitals financial distress across European health systems. In some countries, eg, Poland, no comprehensive data are available, and as public hospitals do not even have the capacity for bankruptcy, hospitals operating with growing debts have been often accepted by the public, while government was forced to launch numerous debt-relief/bail-out programs.^110^ Similar government-led hospital debt-relief programs have also been seen in Romania, Croatia and Hungary in the last two decades.^10^

 Our scoping review identified and mapped a broad spectrum of evidence on the association between hospital FP and Q, helping to build a knowledge base around the topic. There are, however, limitations to be noted. First, only publications in English were considered. Secondly, following guidance on conducting scoping reviews^111^ no quality and risk of bias assessment was conducted for the included studies. The latter limits the ability to formulate policy implications. However, our results provide several implications for future research.

 Firstly, our results help to define research questions for a future systematic and/or realist review. In a few quality areas, we have identified several studies that used similar methods and metrics, thus enabling a meta-analysis of results as part of a future systematic review. For example, among the studies on the association between adverse events and FP, profit per patient and the existence of surgical complications were often used as the main indicators (Table 3). Similarly, in the case of hospital readmissions, most studies used disease specific 30-day readmission indicators and profit variables. Future research can also include a realist review of the association between specific quality dimensions and hospital FP. This type of review aims at explaining the outcomes of complex intervention programmes^112^ by focusing on the relationship between the intervention context and mechanism.^113^ For example, a realist review of programmes focused on limiting hospital readmissions might help to explain the mechanisms by which this area of quality impacts hospital FP (ie, how readmissions impact hospital costs and, in combination with a programme’s financial rewards or penalties, affect the hospital’s overall financial condition).

 Secondly, our results indicate a research gap on the association between hospital FP and Q outside the US healthcare system. There is a need for both methodological/conceptual work on the metrics used to define hospital FP and Q as well as empirical studies to analyse the association between them in non-US healthcare settings. From the perspective of health system administrators and hospital managers, our study indicates the need to plan and implement data reporting systems for both hospital quality and financial aspects that would allow such analyses. One can safely assume that the pressure to contain hospital costs and improve Q will only grow in time, as will the need for this type of research. Even in countries with less resources and lack of adequate data registries, the research on the association between hospital FP and Q can be conducted based on a bottom-up approach, with data gathered via dedicated surveys. It is important to build the knowledge base and scientific evidence to support adequate health policy decisions.

## Conclusion

 This scoping review helps to build a knowledge base around the association between hospital FP and Q. The results suggest that a clear trade-off between these two dimensions is rather unlikely. The existing evidence points in the direction of a positive relationship between quality and hospital FP (in certain contexts, hospitals can simultaneously improve and/or maintain quality and financial profits). The results help define more precise research questions for a future systematic/realistic review while pointing out a potential research gap for both methodological/conceptual work on the metrics used to define hospital FP and Q as well as empirical studies to analyse the association between them in non-US healthcare settings.

## Ethical issues

 Not applicable.

## Competing interests

 Authors declare that they have no competing interests.

## Authors’ contributions

 All authors meet the authorship criteria and agree to the submission of the manuscript. All authors have made substantial contributions to the conception or design of the work, according to the International Committee of Medical Journal Editors (ICMJE) and to the Committee on Publication Ethics (COPE). Conceptualization: KDJ; Methodology: KDJ, EK, MT, and WQ; Acquisition of data: KDJ, EK, MT, OK, and PS; Data analysis: KDJ, EK, MT, OK, PS, RBS, and WQ; Writing-Original Draft Preparation: KDJ, EK, MT, RBS, and WQ; Supervision: KDJ.

## Supplementary files


Supplementary file 1. PRISMA-ScR Checklist.

Supplementary file 2. Search Results Per Database and List of Organizational Websites Screened.

Supplementary file 3. Overview of the Extraction Tables.

Supplementary file 4. List of Included Publications With Full Reference Data Per Type and Year of Publication.

Supplementary file 5. Overview of the Non-empirical Studies.

Supplementary file 6. Overview of the Theoretical Frameworks Used in Empirical Studies.
