# Supplementary material for: The Association Between Hospital Financial Performance and the Quality of Care – A Scoping Literature Review
Source: Int J Health Policy Manag. 2022 Aug 16;11(12):2816–28. doi: 10.34172/ijhpm.2022.6957 (PMC10105205; doi:10.34172/ijhpm.2022.6957)
Supplement: Supplementary file 2 — Search Results Per Database and List of Organizational Websites Screened. [file ijhpm-11-2816-s002.pdf]

Article title: The Association Between Hospital Financial Performance and the Quality of Care – A Scoping Literature Review

Journal name: International Journal of Health Policy and Management (IJHPM)

Authors' information: Katarzyna Dubas-Jakóbczyk[1], Ewa Kocot[1], Marzena Tambor[1], Przemysław Szetela[1], Olga Kostrzewska[2], Richard B. Siegrist Jr[3], Wilm Quentin[4,5]*

[1]Health Economics and Social Security Department, Institute of Public Health, Faculty of Health Sciences, Jagiellonian University Medical College, Krakow, Poland.

[2]Institute of Public Health, Faculty of Health Sciences, Jagiellonian University Medical College, Krakow, Poland.

[3]Harvard T.H. Chan School of Public Health, Boston, MA, USA.

[4]Department of Health Care Management, Technische Universität Berlin, Berlin, Germany.

[5]European Observatory on Health Systems and Policies, WHO European Centre for Health Policy Eurostation (Office 07C020), Brussels, Belgium.

(*Corresponding author: Email: Katarzyna.Dubas@uj.edu.pl)

Supplementary file 2. Search Results Per Database and List of Organizational Websites Screened

Search results per database:

Database: ABI/INFORM
Date of the search: 09/08/2021

Search	Query	Results
S6	((ab(hospital*) OR ab(inpatient*)) AND PEER(yes)) AND (((ab(financial performance) OR ab(financial standing) OR ab(financial situation) OR ab(financial indicator*) OR ab(financial condition*) OR ab(profit*) OR ab(operating margin*) OR ab(debt*) OR ab(financial failure) OR ab(financial distress)) AND PEER(yes)) OR ((ab(financial measure*) OR ab(financial parameter*) OR ab(liquidity) OR ab(asset turnover) OR ab(cash flow)) AND PEER(yes))) AND ((ab(quality) OR ab(staff*) OR ab(technology) OR ab(patient* safety) OR ab(adverse event*) OR ab(health outcome*) OR ab(readmission*) OR ab(complication*) OR ab(patient* satisfaction)) AND PEER(yes))	644
S5	ab(quality) OR ab(staff*) OR ab(technology) OR ab (patient* safety) OR ab (adverse event*) OR ab (health outcome*) OR ab(readmission*) OR ab(complication*) OR ab (patient* satisfaction)	213155
S4	((ab (financial performance) OR ab (financial standing) OR ab (financial situation) OR ab (financial indicator*) OR ab(financial condition*) OR ab(profit*) OR ab(operating margin*) OR ab(debt*) OR ab(financial failure) OR ab(financial distress)) AND PEER(yes)) OR ((ab(financial measure*) OR ab(financial parameter*) OR ab(liquidity) OR ab(asset turnover) OR ab(cash flow)) AND PEER(yes))	78731
S3	ab (financial measure*) OR ab (financial parameter*) OR ab(liquidity) OR ab (asset turnover) OR ab (cash flow)	23739

S2	ab (financial performance) OR ab (financial standing) OR ab (financial situation) OR ab (financial indicator*) OR ab (financial condition*) OR ab(profit*) OR ab (operating margin*) OR ab(debt*) OR ab (financial failure) OR ab(financial distress)	65268
S1	ab(hospital*) OR ab(inpatient*)	24893

Database: Business Source Complete
Date of the search: 04/08/2021

Only in English

Expanders - Apply equivalent subjects

Search	Query	Actions
#30	S3 AND S19 AND S29	1,703
#29	S20 OR S21 OR S22 OR S23 OR S24 OR S25 OR S26 OR S27 OR S28	1,606,010
#28	AB complication*	10,414
#27	AB adverse event*	3,779
#26	AB readmission*	1,342
#25	AB patient* satisfaction	3,715
#24	AB patient* safety	8,662
#23	AB health outcome*	16,973
#22	AB technology	1,062,141
#21	AB staff*	172,312
#20	AB quality	394,211
#19	S4 OR S5 OR S6 OR S7 OR S8 OR S9 OR S10 OR S11 OR S12 OR S13 OR S14 OR S15 OR S16 OR S17 OR S18	876,981
#18	AB asset turnover	1,514
#17	AB liquidity	45,642
#16	AB debt*	214,459
#15	AB cash flow	31,823
#14	AB operating margin*	7,527
#13	AB profit*	411,580
#12	AB financial parameter*	3,866
#11	AB financial measure*	36,858
#10	AB financial distress	3,370
#9	AB financial failure	15,161
#8	AB financial condition*	35,319
#7	AB financial indicator*	11,872
#6	AB financial situation	12,755
#5	AB financial standing	2,742
#4	AB financial performance	138,856
#3	S1 OR S2	181,840
#2	AB inpatient*	5,615
#1	AB hospital*	179,871

Database: EconLit
Date of the search: 04/08/2021

Only in English

Expanders - Apply equivalent subjects

Search	Query	Actions
#30	S3 AND S19 AND S29	322
#29	S20 OR S21 OR S22 OR S23 OR S24 OR S25 OR S26 OR S27 OR S28	112,517
#28	AB adverse event*	912
#27	AB readmission*	790
#26	AB patient* satisfaction	186
#25	AB patient* safety	214
#24	AB mortality	207
#23	AB health outcome*	6,331
#22	AB technology	52,672
#21	AB staff*	4,568
#20	AB quality	53,382
#19	S4 OR S5 OR S6 OR S7 OR S8 OR S9 OR S10 OR S11 OR S12 OR S13 OR S14 OR S15 OR S16 OR S17 OR S18	121,925
#18	AB asset turnover	468
#17	AB liquidity	15,863
#16	AB debt*	28,692
#15	AB cash flow	6,074
#14	AB operating margin*	690
#13	AB profit*	43,133
#12	AB financial parameter*	3,093
#11	AB financial measure*	16,206
#10	AB financial distress	2,076
#9	AB financial failure	3,175
#8	AB financial condition*	11,391
#7	AB financial indicator*	4,851
#6	AB financial situation	2,954
#5	AB financial standing	339
#4	AB financial performance	12,601
#3	S1 OR S2	6605
#2	AB inpatient*	826
#1	AB hospital*	6,759

Database: Embase via Ovid
Date of the search: 10/08/2021

Set	Query	Results
#4	1 and 2 and 3	3126
#3	(quality or staff* or technology or health outcome* or patient* safety or adverse event* or readmission* or complication* or patient* satisfaction). ab.	3395310
#2	(financial performance or financial situation or financial standing or financial indicator* or financial condition* or profit* or operating margin* or debt* or financial measure* or financial parameter* or financial failure or financial distress or liquidity or asset turnover or cash flow). ab.	40859
#1	(hospital* or inpatient*). ab.	2073729

Database: Medline via PubMed
Date of the search: 09/08/2021

Search	Query	Results
#33	Search: ((((hospital*[Title/Abstract]) OR (inpatient*[Title/Abstract])) OR (administration, hospital[MeSH])) AND ((((((((((((((financial performance[Title/Abstract]) OR (financial standing[Title/Abstract])) OR (financial situation[Title/Abstract])) OR (financial indicator*[Title/Abstract])) OR (financial condition*[Title/Abstract])) OR (financial failure[Title/Abstract])) OR (financial distress[Title/Abstract])) OR (financial measure*[Title/Abstract])) OR (financial parameter*[Title/Abstract])) OR (profit*[Title/Abstract])) OR (operating margin*[Title/Abstract])) OR (debt*[Title/Abstract])) OR (liquidity[Title/Abstract])) OR (asset turnover[Title/Abstract])) OR (cash flow[Title/Abstract]))) AND ((((((((((quality[Title/Abstract]) OR (assessment, healthcare quality[MeSH])) OR (staff*[Title/Abstract])) OR (technology[Title/Abstract])) OR (health outcome*[Title/Abstract])) OR (patient* safety[Title/Abstract])) OR (readmission*[Title/Abstract])) OR (adverse event*[Title/Abstract])) OR (complication*[Title/Abstract])) OR (patient* satisfaction[Title/Abstract])) Filters: English	2,526
#32	Search: ((((hospital*[Title/Abstract]) OR (inpatient*[Title/Abstract])) OR (administration, hospital[MeSH])) AND ((((((((((((((financial performance[Title/Abstract]) OR (financial standing[Title/Abstract])) OR (financial situation[Title/Abstract])) OR (financial indicator*[Title/Abstract])) OR (financial condition*[Title/Abstract])) OR (financial failure[Title/Abstract])) OR (financial distress[Title/Abstract])) OR (financial measure*[Title/Abstract])) OR (financial parameter*[Title/Abstract])) OR (profit*[Title/Abstract])) OR (operating margin*[Title/Abstract])) OR (debt*[Title/Abstract])) OR (liquidity[Title/Abstract])) OR (asset turnover[Title/Abstract])) OR (cash flow[Title/Abstract]))) AND ((((((((((quality[Title/Abstract]) OR (assessment, healthcare quality[MeSH])) OR (staff*[Title/Abstract])) OR (technology[Title/Abstract])) OR (health outcome*[Title/Abstract])) OR (patient* safety[Title/Abstract])) OR (readmission*[Title/Abstract])) OR (adverse event*[Title/Abstract])) OR (complication*[Title/Abstract])) OR (patient* satisfaction[Title/Abstract]))	2,765
#31	Search: (((((((((quality [Title/Abstract]) OR (assessment, healthcare quality [MeSH])) OR (staff*[Title/Abstract])) OR (technology [Title/Abstract])) OR (health outcome*[Title/Abstract])) OR (patient* safety [Title/Abstract])) OR (readmission*[Title/Abstract])) OR (adverse event*[Title/Abstract])) OR (complication*[Title/Abstract])) OR (patient* satisfaction [Title/Abstract])	3,242,709
#30	Search: patient* satisfaction [Title/Abstract]	101,574
#29	Search: complication* [Title/Abstract]	1,055,851
#28	Search: adverse event* [Title/Abstract]	182,180
#27	Search: readmission* [Title/Abstract]	34,620
#26	Search: patient* safety [Title/Abstract]	333,191
#25	Search: health outcome* [Title/Abstract]	61,285
#24	Search: technology [Title/Abstract]	363,169

#23	Search: staff* [Title/Abstract]	182,813
#22	Search: assessment, healthcare quality [Mesh]	341,311
#21	Search: quality [Title/Abstract]	1,143,604
#20	Search: ((((((((((((((financial performance[Title/Abstract]) OR (financial standing[Title/Abstract])) OR (financial situation[Title/Abstract])) OR (financial indicator*[Title/Abstract])) OR (financial condition*[Title/Abstract])) OR (financial failure[Title/Abstract])) OR (financial distress[Title/Abstract])) OR (financial measure* [Title/Abstract])) OR (financial parameter*[Title/Abstract])) OR (profit*[Title/Abstract])) OR (operating margin*[Title/Abstract])) OR (debt*[Title/Abstract])) OR (liquidity[Title/Abstract])) OR (asset turnover[Title/Abstract])) OR (cash flow[Title/Abstract])	
#19	Search: cash flow [Title/Abstract]	597
#18	Search: asset turnover [Title/Abstract]	11
#17	Search: liquidity [Title/Abstract]	368
#16	Search: debt* [Title/Abstract]	5,436
#15	Search: operating margin*[Title/Abstract]	254
#14	Search: profit*[Title/Abstract]	28,133
#13	Search: financial parameter*[Title/Abstract]	40
#12	Search: financial measure*[Title/Abstract]	93
#11	Search: financial distress [Title/Abstract]	358
#10	Search: financial failure [Title/Abstract]	17
#9	Search: financial condition*[Title/Abstract]	347
#8	Search: financial indicator*[Title/Abstract]	131
#7	Search: financial situation [Title/Abstract]	900
#6	Search: financial standing [Title/Abstract]	46
#5	Search: financial performance [Title/Abstract]	986
#4	Search: (hospital*[Title/Abstract]) OR (inpatient*[Title/Abstract])	1,628,345
#3	Search: administration, hospital [Mesh]	270,147
#2	Search: inpatient*[Title/Abstract]	131,855
#1	Search: hospital*[Title/Abstract]	1413276

Database: Scopus
Date of the search: 09/08/2021

Search	Query	Actions
#31	ABS (hospital*)) OR (ABS (inpatient*))) AND ((ABS (financial AND performance)) OR (ABS (financial AND standing)) OR (ABS (financial AND situation)) OR (ABS (financial AND indicator*)) OR (ABS (financial AND condition*)) OR (ABS (financial AND failure)) OR (ABS (financi al AND distress)) OR (ABS (financial AND measure*)) OR (ABS (financial AN D parameter*)) OR (ABS (profit*)) OR (ABS (operating AND margin*)) OR (A BS (debt*)) OR (ABS (liquidity)) OR (ABS (asset AND turnover)) OR (ABS (cash AND f low)) AND ((ABS (quality)) OR (ABS (staff*)) OR (ABS (technology)) OR (AB S (health AND outcome*)) OR (ABS (patient* AND safety)) OR (ABS (readmission*)) OR (ABS (adverse AN	6,753

	D event*)) OR (ABS (complication*)) OR (ABS (patient* AND satisfaction))) AND (LIMIT-TO (PUBSTAGE, "final")) AND (LIMIT-TO (DOCTYPE, "ar") OR LIMIT-TO (DOCTYPE, "re") OR LIMIT-TO (DOCTYPE, "bk")) AND (LIMIT-TO (LANGUAGE, "English"))	
#30	ABS (hospital*) OR (ABS (inpatient*)) AND ((ABS (financial AND performance)) OR (ABS (financial AND standing)) OR (ABS (financial AND situation)) OR (ABS (financial AND indicator*)) OR (ABS (financial AND condition*)) OR (ABS (financial AND failure)) OR (ABS (financial AND distress)) OR (ABS (fin ancial AND measure*)) OR(ABS (financial AND parameter*)) OR (ABS (profit *)) OR (ABS (operating AND margin*)) OR (ABS (debt*)) OR (ABS (liquidity) OR (ABS (asset AND turnover))OR (ABS (cash AND flow))) AND ((ABS (qu ality)) OR (ABS (staff*)) OR (ABS (technology)) OR (ABS (health AND outco me*))OR (ABS (patient* AND safety)) OR (ABS (readmission*)) OR (ABS (adverse AND event*)) OR (ABS (complication*)) OR (ABS (patient* AND satisfaction)))	8,420
#29	ABS (quality)) OR (ABS (staff*)) OR (ABS (technology)) OR (ABS (health AND outcome*)) OR (ABS (patient* AND safety)) OR (ABS (readmission*)) OR (ABS (adverse AND event*)) OR (ABS (complication*)) OR (ABS (patient* AND satisfaction))	7,638,569
#28	ABS patient* AND satisfaction	103,653
#27	ABS complication*	1,089,251
#26	ABS adverse event*	241,405
#25	ABS readmission*	31,951
#24	ABS patient* AND safety	337,845
#23	ABS health AND outcome*	392,168
#22	ABS technology	2,807,192
#21	ABS staff*	320,959
#20	ABS quality	3,210,443
#19	ABS (financial AND performance))OR (ABS (financial AND standing)) OR AB S (financial AND situation)) OR (ABS (financial AND indicator*)OR (ABS (fina ncial AND condition*)) OR (ABS (financial AND failure)) OR (ABS (financial AND distress)) OR (ABS (financial AND measure *)) OR (ABS (financial AND parameter*)) OR (ABS (profit*)) OR (ABS (operati ng AND margin*)) OR (ABS (debt*)) OR (ABS (liquidity)) OR (ABS (asset AND turnover)) OR (ABS(cash AND flow))	494,422
#18	ABS cash AND flow	16,857
#17	ABS asset AND turnover	1,302
#16	ABS liquidity	18,989
#15	ABS debt*	48,635
#14	ABS operating AND margin*	16,183
#13	ABS profit*	250,908
#12	ABS financial AND parameter*	13,979
#11	ABS financial AND measure*	63,560
#10	ABS financial AND distress	5,372
#9	ABS financial AND failure	13,782
#8	ABS financial AND condition*	43,265

#7	ABS financial AND indicator*	18,572
#6	ABS financial AND situation	20,803
#5	ABS financial AND standing	1,898
#4	ABS financial AND performance	57,446
#3	ABS (hospital*)) OR (ABS inpatient*))	1,611,008
#2	ABS inpatient*	127,785
#1	ABS hospital*	1,553,321

Database: Web of Science
Date of the search: 09/08/2021

Set	Query	Results
#32	#3 AND #19 AND #29 and Articles or Review Articles or Book Chapters (Document Types) and English (Languages)	5,322
#31	#3 AND #19 AND #29 and Articles or Review Articles or Book Chapters (Document Types)	5,765
#30	#3 AND #19 AND #29	6,059
#29	#20 OR #21 OR #22 OR #23 OR #24 OR #25 OR #26 OR #27 OR #28	5,188,373
#28	AB=patient* satisfaction	81,170
#27	AB=complication*	749,409
#26	AB=adverse event*	207,970
#25	AB=patient* safety	266,958
#24	AB=readmission*	27,601
#23	AB=health outcome*	333,550
#22	AB=technology	1,712,040
#21	AB=staff*	198,370
#20	AB=quality	2,276,750
#19	#4 OR #5 OR #6 OR #7 OR #8 OR #9 OR #10 OR #11 OR #12 OR #13 OR #14 OR #15 OR #16 OR #17 OR #18	353,941
#18	AB=cash flow	11,134
#17	AB=asset turnover	1,079
#16	AB=liquidity	15,005
#15	AB=debt*	34,046
#14	AB=operating margin	16,062
#13	AB=profit*	167,525
#12	AB= financial parameter*	10,708
#11	AB= financial measure*	49,311
#10	AB= financial distress	4,888
#9	AB= financial failure	9,403
#8	AB=financial condition*	33,342
#7	AB=financial indicator*	15,997
#6	AB=financial situation	16,553
#5	AB=financial standing	3,547
#4	AB=financial performance	42,799
#3	#1 OR #2	1,094,770
#2	AB=inpatient*	99,089
#1	AB=hospital*	1,050,541

Websites screening:

Organization	www	Screening date
WHO – World Health Organization: Health	https://www.who.int/health-topics	24/08/2021
OECD:Health Care Quality and Outcomes	https://www.oecd.org/health/health-systems/health-care-quality-and-outcomes.htm	24/08/2021
HOPE – European Hospital and Healthcare Federation	https://hope.be	24/08/2021
RAND Corporation	https://www.rand.org	24/08/2021
NICE – National Institute for Health and Care Excellence	https://www.nice.org.uk	24/08/2021
AHA – American Hospital Association	https://www.aha.org	26/08/2021
AHRQ – Agency for Healthcare Research and Quality	https://www.ahrq.gov	24/08/2021
NSQHS – National Safety and Quality Health Service	https://www.safetyandquality.gov.au	26/08/2021
HIMMS – Healthcare Information and Management System Society	https://www.himss.org	26/08/2021
PIIE – Peterson Institute for International Economics	https://www.piie.com	24/08/2021
ISQua – International Society for Quality in Healthcare Care	https://isqua.org	26/08/2021
AHD – American Hospital Directory	https://www.ahd.com	26/08/2021
AHIMA – American Health Information Management Association	https://www.ahima.org	30/08/2021
America’s Essential Hospitals	https://essentialhospitals.org	30/08/2021
NPSF – National Patient Safety Foundation	https://npsf.digitellinc.com/npsf/	30/08/2021
NCQA – National Committee for Quality Assurance	https://www.ncqa.org	30/08/2021
FAH - Federation of American Hospitals	https://www.fah.org	30/08/2021
Eurostat: Health	https://ec.europa.eu/eurostat/web/health	30/08/2021
Care Quality Commission	https://www.cqc.org.uk	30/08/2021
Care Inspectorate	https://www.careinspectorate.com	30/08/2021
IHI – Institute for Healthcare Improvement	http://www.ihi.org	30/08/2021
