# Supplementary material for: The Association Between Hospital Financial Performance and the Quality of Care – A Scoping Literature Review
Source: Int J Health Policy Manag. 2022 Aug 16;11(12):2816–28. doi: 10.34172/ijhpm.2022.6957 (PMC10105205; doi:10.34172/ijhpm.2022.6957)
Supplement: Supplementary file 3 — Overview of the Extraction Tables. [file ijhpm-11-2816-s003.pdf]

Article title: The Association Between Hospital Financial Performance and the Quality of Care – A Scoping Literature Review

Journal name: International Journal of Health Policy and Management (IJHPM)

Authors' information: Katarzyna Dubas-Jakóbczyk[1], Ewa Kocot[1], Marzena Tambor[1], Przemysław Szetela[1], Olga Kostrzewska[2], Richard B. Siegrist Jr[3], Wilm Quentin[4,5]*

[1]Health Economics and Social Security Department, Institute of Public Health, Faculty of Health Sciences, Jagiellonian University Medical College, Krakow, Poland.

[2]Institute of Public Health, Faculty of Health Sciences, Jagiellonian University Medical College, Krakow, Poland.

[3]Harvard T.H. Chan School of Public Health, Boston, MA, USA.

[4]Department of Health Care Management, Technische Universität Berlin, Berlin, Germany.

[5]European Observatory on Health Systems and Policies, WHO European Centre for Health Policy Eurostation (Office 07C020), Brussels, Belgium.

(*Corresponding author: Email: Katarzyna.Dubas@uj.edu.pl)

Supplementary file 3. Overview of the Extraction Tables

Data extraction and coding table for empirical studies:

Research question	Data extracted	Coding examples
RQ1	authors/title	N/A
	year of publication	• before 1990<br>• 1990 – 1999<br>• 2000 – 2009<br>• 2010 – 2021
	research country	N/A (list of countries)
	research design	• quantitative (longitudinal vs. cross-sectional)<br>• qualitative
	characteristics of hospitals	• sample: regional vs. national; case study<br>• number of hospitals, period of data collection
RQ2	conceptual/theoretical framework	N/A (framework description)
RQ3	type of association being assessed	• statistical method used<br>• control variables used<br>• impact of FP on QoC (FP as predictor variable)<br>• impact of QoC on FP (QoC as predictor variable)<br>• both directions
RQ4	financial performance definition and measures	• number of indicators; single indicators vs composite measures<br>• level of FP measurement (hospital vs. patient / procedure)<br>• profitability (diverse measures of profit, and return on assets, equity etc.)<br>• liquidity (e.g., current ratio)

		• debt management (e.g., debt ratio)<br>• asset management (e.g., asset turnover)<br>• other
RQ5	quality of care definition and measures	• number of indicators; single indicators vs composite measures<br>• quality area (e.g. patient satisfaction, adverse events, readmissions, mortality, etc).<br>• structure (input indicators – resources used)<br>• process (indicators related to care delivery)<br>• outcome (intermediate and final health outcomes)
RQ6	identified association	• result of statistical analysis (ratio, statistical significance)<br>• overall assessment of the association between FP and QoC<br>○ positive<br>○ negative<br>○ lack of association<br>○ mixed results
RQ7	limitations stated	• related to data<br>• related to methods<br>• other

Data extraction and coding table for other type of publications:

Research question	Data extracted	Coding examples
RQ1	authors/title	N/A
	year of publication	• before 1990<br>• 1990 – 1999<br>• 2000 – 2009<br>• 2010 – 2021
	type of study	• theoretical paper<br>• systematic review<br>• dissertation<br>• technical paper
	objective	N/A (description)
	results	N/A (description)
	conclusions	N/A (description)
