# Supplementary material for: The Association Between Hospital Financial Performance and the Quality of Care – A Scoping Literature Review
Source: Int J Health Policy Manag. 2022 Aug 16;11(12):2816–28. doi: 10.34172/ijhpm.2022.6957 (PMC10105205; doi:10.34172/ijhpm.2022.6957)
Supplement: Supplementary file 4 — List of Included Publications With Full Reference Data Per Type and Year of Publication. [file ijhpm-11-2816-s004.pdf]

Article title: The Association Between Hospital Financial Performance and the Quality of Care – A Scoping Literature Review

Journal name: International Journal of Health Policy and Management (IJHPM)

Authors' information: Katarzyna Dubas-Jakóbczyk[1], Ewa Kocot[1], Marzena Tambor[1], Przemysław Szetela[1], Olga Kostrzewska[2], Richard B. Siegrist Jr[3], Wilm Quentin[4,5]*

[1]Health Economics and Social Security Department, Institute of Public Health, Faculty of Health Sciences, Jagiellonian University Medical College, Krakow, Poland.

[2]Institute of Public Health, Faculty of Health Sciences, Jagiellonian University Medical College, Krakow, Poland.

[3]Harvard T.H. Chan School of Public Health, Boston, MA, USA.

[4]Department of Health Care Management, Technische Universität Berlin, Berlin, Germany.

[5]European Observatory on Health Systems and Policies, WHO European Centre for Health Policy Eurostation (Office 07C020), Brussels, Belgium.

(*Corresponding author: Email: Katarzyna.Dubas@uj.edu.pl)

Supplementary file 4. List of Included Publications With Full Reference Data Per Type and Year of Publication

List of included studies per year and type of publication (ordered by the publication year):

No	Reference number	First author/s and publication year	Full reference	Year of publication	Country (for empirical studies) / Publication type (for other)
1	49	**Harkey & Vraciu 1992**	Harkey J, Vraciu R. Quality of health care and financial performance: is there a link? *Health Care Management Review*. 1992;17(4):55–63.	1992	US
2	92	**Hsia & Ahern 1992**	Hsia DC, Ahern CA. Good quality care increases hospital profits under prospective payment. *Health Care Financing Review*. 1992;13(3):17–26.	1992	US
3	87	**Cleverley et al. 1992**	Cleverley WO, Harvey RK. Is there a link between hospital profit and quality? Healthcare Financial Management: *Journal of the Healthcare Financial Management Association.* 1992;46(9):40,42,44-45.	1992	US
4	61	**Nelson et al. 1992**	Nelson EC, Rust RT, Zahorik A, Rose RL, Batalden P, Siemanski BA. Do patient perceptions of quality relate to hospital financial performance? *Journal of Health Care Marketing.* 1992;12(4):6–13.	1992	US
5	31	**Fleming & Boles 1994**	Fleming ST, Boles KE. Financial and clinical performance: Bridging the gap. *Health Care Management Review.* 1994;19(1):11–17.	1994	theoretical, conceptual paper
6	95	**Langland-Orban et al. 1996**	Langland-Orban B, Gapenski LC, Vogel WB. Differences in characteristics of hospitals with sustained high and sustained low profitability. *Hospital & Health Services Administration*. 1996;41(3):385–401.	1996	US

**7**	**55**	**Irwin et al. 1998**	Irwin JG, Hoffman JJ, Lamont BT. The effect of the acquisition of technological innovations on organizational performance: A resource-based view. *Journal of* Engineering *and Technology Management - JET-M.* 1998;15(1):25–54.	1998	US
**8**	**57**	**Li & Collier 2000**	Li LX, Collier DA. The role of technology and quality on hospital financial performance: An exploratory analysis. *International Journal of Service Industry Management.* 2000;11(3):202–224.	2000	US
**9**	**63**	**Parente et al. 2001**	Parente ST, Dunbar JL. Is health information technology investment related to the financial performance of US hospitals? An exploratory analysis. *International Journal of Healthcare Technology and Management.* 2001;3(1):48–58.	2001	US
**10**	**60**	**McCue et al. 2003**	McCue M, Mark BA, Harless DW. Nurse staffing, quality, and financial performance. *Journal of Health Care Finance.* 2003;29(4):54–76.	2003	US
**11**	**72**	**Encinosa & Bernard 2005**	Encinosa WE, Bernard DM. Hospital finances and patient safety outcomes. Inquiry: *A Journal of Medical Care Organization, Provision and Financing.* 2005; 42(1):60–72.	2005	US
**12**	**16**	**Beauvais & Wells 2006**	Beauvais B, Wells R. Does money really matter? A review of the literature on the relationships between healthcare organization finances and quality. *Hosp Top*. 2006;84(2):20-28. doi:10.3200/htps.84.2.20-29.	2006	literature review
**13**	**44**	**Dimick et al. 2006**	Dimick JB, Weeks WB, Karia RJ, Das S, Campbell DAJ. Who pays for poor surgical quality? Building a business case for quality improvement. *Journal of the American College of Surgeons.* 2006;202(6):933–937.	2006	US
**14**	**77**	**Menachemi et al. 2006**	Menachemi N, Burkhardt J, Shewchuk R, Burke D, Brooks RG. Hospital information technology and positive financial performance: a different approach to finding an ROI. J Healthc Manag. 2006 Jan-Feb;51(1):40-58; discussion 58-9. PMID: 16479749.	2006	US
**15**	**37**	**Alexander et al. 2006**	Alexander JA, Weiner BJ, Griffith J. Quality improvement and hospital financial performance. *Journal of Organizational Behavior.* 2006;27(7):1003–1029. doi: 0.1002/job.401	2006	US
**16**	**53**	**Hegji 2006**	Hegji C. Correlates of Hospital Quality: A Preliminary Study. *Economics Bulletin.* 2006;9(4):1–13.	2006	US
**17**	**89**	**Cowan et al. 2006**	Cowan MJ, Shapiro M, Hays RD, et al. The effect of a multidisciplinary hospitalist/physician and advanced practice nurse collaboration on hospital costs. *The Journal of Nursing Administration.* 2006;36(2):79–85.	2006	US
**18**	**46**	**Englesbe et al. 2006**	Englesbe MJ, Dimick J, Mathur A, et al. Who pays for biliary complications following liver transplant? A business case for quality improvement. *Am J Transplant.* 2006 Dec;6(12):2978-82. doi:10.1111/j.1600-6143.2006.01575.x. PMID: 17294525.	2006	US
**19**	**82**	**Bazzoli et al. 2007**	Bazzoli GJ, Clement JP, Lindrooth RC, et al. Hospital financial condition and operational decisions related to the quality of hospital care. *Medical Care Research and Review: MCRR.* 2007;64(2):148–168.	2007	US
**20**	**74**	**Kazley & Ozcan 2007**	Kazley AS, Ozcan YA. Organizational and Environmental Determinants of Hospital EMR Adoption: A National Study. *J Med Syst* 31. 2007;375–384. doi:10.1007/s10916-007-9079-7	2007	US
**21**	**51**	**Hegji & Self 2007**	Hegji, C. E., Self, D. R., & Findley, C. S. C. (2007). The link between hospital quality and services profitability. International Journal of Pharmaceutical and Healthcare Marketing, 1(4), 290–303.	2007	US
**22**	**38**	**Ammori et al. 2007**	Ammori JB, Pelletier SJ, Lynch R, Cohn J, Ads Y, Campbell DA, Englesbe MJ. Incremental costs of post-liver transplantation complications. *J Am Coll Surg*. 2008 Jan;206(1):89-95. doi:10.1016/j.jamcollsurg.2007.06.292. Epub 2007 Sep 18. PMID: 18155573.	2007	US

23	96	**Pirson et al. 2008**	Pirson M, Leclercq P, Jackson T, Leclercq M, Garrino M, Sion C. Financial consequences of hospital-acquired bacteraemia in three Belgian hospitals in 2003 and 2004. *The Journal of Hospital Infection.* 2008;68(1):9–16.	2008	Belgium
24	86	**Bazzoli et al. 2008**	Bazzoli GJ, Chen HF, Zhao M, Lindrooth RC. Hospital financial condition and the quality of patient care. *Health Economics.* 2008;17(8):977–995.	2008	US
25	39	**Ammori et al. 2008**	Ammori JB, Pelletier SJ, Mathur A, et al. Financial implications of surgical complications in pediatric liver transplantation. *Pediatric Transplantation*. 2008; 12(2):174–179.	2008	US
26	70	**Zhao et al. 2008**	Zhao M, Bazzoli GJ, Clement JP, Lindrooth RC, Nolin JM, Chukmaitov AS. Hospital Staffing Decisions: Does Financial Performance Matter? INQUIRY: *The Journal of Health Care Organization, Provision, and Financing*. August 2008;293-307. doi:10.5034/inquiryjrnl_45.03.293	2008	US
27	59	**Maiga & Jacobs 2009**	Maiga AS, Jacobs FA. Leadership, nonfinancial, and financial outcomes: The case of community hospitals. *Accounting and the Public Interest*. 2009;9(1):166–190. doi:10.2308/api.2009.9.1.166	2009	US
28	52	**Hegji & Self 2009**	Hegji CE, Self DR. The impact of hospital quality on profits, volume, and length of stay. *Health Marketing Quarterly.* 2009;26(3):209–223.	2009	US
29	66	**Self et al. 2010**	Self DR, Hegji CE, Self RM. Alternative quality measures and profitability of hospital inpatient services offered. *Journal of Hospital Marketing & Public Relations.* 2010;20(1):2–13.	2010	US
30	54	**Ho et. al. 2010**	Ho D, Lynch RJ, Ranney DN, Magar A, Kubus J, Englesbe MJ. Financial impact of surgical site infection after kidney transplantation: implications for quality improvement initiative design. *J Am Coll Surg*. 2010 Jul;211(1):99-104.	2010	US
31	32	**Holt et al. 2011**	Holt HD, Clark J, DelliFraine J, Brannon D. Organizing for performance: what does the empirical literature reveal about the influence of organizational factors on hospital financial performance? *Advances in Health Care Management.* 2011;11:21–62.	2011	literature review
32	73	**Ginn et al. 2011**	Ginn GO, Shen JJ, Moseley CB. Hospital financial position and the adoption of electronic health records. *J Healthc Manag*. 2011 Sep-Oct;56(5):337-50; discussion 351-2. PMID: 21991681.	2011	US
33	76	**Ly et al. 2011**	Ly DP, Jha AK, Epstein AM. The association between hospital margins, quality of care, and closure or other change in operating status. *J Gen Intern Med*. 2011 Nov;26(11):1291-6. doi: 10.1007/s11606-011-1815-5.	2011	US
34	35	**Cusack 2012**	Cusack S. The Impact of Quality on Financial Performance of Acute Care Hospitals. University of the Sciences in Philadelphia. 2012	2012	PhD dissertation
35	80	**Shen & Ginn 2012**	Shen JJ, Ginn GO. Financial position and adoption of electronic health records: a retrospective longitudinal study. *J Health Care Finance*. 2012 Spring;38(3):61-77. PMID: 22515045.	2012	US
36	79	**Navathe et al. 2012**	Navathe AS, Volpp KG, Konetzka RT, et al. A longitudinal analysis of the impact of hospital service line profitability on the likelihood of readmission. *Medical Care Research and Review*: *MCRR*. 2012;69(4):414–431.	2012	US
37	64	**Reiter et al. 2012**	Reiter KL, Harless DW, Pink GH, Mark BA. Minimum nurse staffing legislation and the financial performance of California hospitals. *Health Services Research.* 2012;47(3 Pt 1):1030–1050.	2012	US
38	45	**Eappen et al. 2013**	Eappen S, Lane BH, Rosenberg B, et al. Relationship between occurrence of surgical complications and hospital finances. *JAMA.* 2013 Apr 17;309(15):1599-606. doi:10.1001/jama.2013.2773. PMID: 23592104.	2013	US
39	75	**Lindrooth et al. 2013**	Lindrooth RC, Konetzka RT, Navathe AS, Zhu J, Chen W, Volpp K. The impact of profitability of hospital admissions on mortality. *Health Serv Res*. 2013;48(2 Pt 2):792-809. doi:10.1111/1475-6773.12026	2013	US

**40**	**47**	**Everhart et al. 2013**	Everhart D, Neff D, Al-Amin M, Nogle J, Weech-Maldonado R. The effects of nurse staffing on hospital financial performance: competitive versus less competitive markets. *Health Care Management Review.* 2013;38(2):146–155.	2013	US
**41**	**33**	**Zengul et al. 2014**	Zengul FD, Weech-Maldonado R, Savage GT. Technological innovations, and hospital performance: a systematic review of the literature. *INNOVATION AND ENTREPRENEURSHIP IN HEALTH.* 2014;1:13–26.	2014	literature review
**42**	**93**	**Jenks et al. 2014**	Jenks PJ, Laurent M, McQuarry S, Watkins R. Clinical and economic burden of surgical site infection (SSI) and predicted financial consequences of elimination of SSI from an English hospital. *Journal of Hospital Infection. 2014;*86(1):24–33.	2014	UK
**43**	**43**	**Clement et al. 2014**	Clement RC, Kheir MM, Derman PB, et al. What are the economic consequences of unplanned readmissions after TKA? *Clinical Orthopaedics and Related Research.* 2014;472(10):3134–3141.	2014	US
**44**	**48**	**Flynn et al. 2014**	Flynn DN, Speck RM, Mahmoud NN, David G, Fleisher LA. The impact of complications following open colectomy on hospital finances: a retrospective cohort study. *PERIOPERATIVE MEDICINE, 3*. doi:1186/2047-0525-3-1	2014	US
**45**	**56**	**Kodera & Yoneda 2015**	Kodera T, Yoneda K. Hospital accreditation and financial conditions of public hospitals: Evidence from hospitals in Chubu. *Journal of Economics and Economic Education Research.* 2015;16(3):97–106.	2015	Japan
**46**	**71**	**Dong 2015**	Dong GN. Performing well in financial management and quality of care: evidence from hospital process measures for treatment of cardiovascular disease. *BMC Health Serv Res.* 2015;15:45. Published 2015 Feb 1. doi:10.1186/s12913-015-0690-x	2015	US
**47**	**84**	**Turner et al. 2015**	Turner JS, Broom KD, Counte MA. Is There a Relationship Between Value-Based Purchasing and Hospital Profitability? An Exploratory Study of Missouri Hospitals. *Health Services Research and Managerial Epidemiology.* 2015	2015	US
**48**	**34**	**Oner et al. 2016**	Oner N, Zengul FD, Ozaydin B, et al. Organizational and Environmental Factors Associated with Hospital Financial Performance: A Systematic Review. *Journal of Health Care Finance.* 2016;43(2):3-37.	2016	literature review
**49**	**50**	**Healy et al. 2016**	Healy MA, Mullard AJ, Campbell DAJ, Dimick JB. Hospital and Payer Costs Associated with Surgical Complications. *JAMA Surgery.* 2016;151(9):823–830.	2016	US
**50**	**88**	**Collum et al. 2016**	Collum TH, Menachemi N, Sen B. Does electronic health record use improve hospital financial performance? Evidence from panel data. *Health Care Management Review*. 2016;41(3):267–274. doi: 10.1097/HMR.0000000000000068.	2016	US
**51**	**69**	**Wright et al. 2016**	Wright JD, Tergas AI, Hou JY, et al. Effect of Regional Hospital Competition and Hospital Financial Status on the Use of Robotic-Assisted Surgery. *JAMA Surgery.* 2016;151(7):612–620.	2016	US
**52**	**62**	**Nevola et al. 2016**	Nevola A, Pace C, Karim SA, Morris ME. Revisiting "the determinants of hospital profitability" in Florida. *Journal of Health Care Finance.* 2016;43(2):38–60.	2016	US
**53**	**83**	**Nguyen et al. 2016**	Nguyen OK, Halm EA, Makam AN. Relationship between hospital financial performance and publicly reported outcomes. *J Hosp Med.* 2016;11(7):481–488. doi:10.1002/jhm.2570	2016	US
**54**	**15**	**Barnes et al. 2017**	Barnes M, Oner N, Ray MN, Zengul FD. Exploring the Association between Quality and Financial Performance in U.S. Hospitals: A Systematic Review. *Journal of Health Care Finance*. Vol. 44, No. 2, FALL 2017.	2017	literature review
**55**	**65**	**Richter & Muhlestein 2017**	Richter JP, Muhlestein DB. Patient experience and hospital profitability: Is there a link? Health Care Manage Rev. 2017 Jul/Sep;42(3):247-257. doi: 10.1097/HMR.0000000000000105. PMID: 27050925.	2017	US

**56**	**90**	**Crowe et al. 2017**	Crowe D, Garman AN, Li C-C, Helton J, Anderson MM, Butler P. Leadership development practices and hospital financial outcomes. *Health Services Management Research.* 2017;30(3):140–147.	2017	US
**57**	**68**	**Wang et al. 2018**	Wang T, Wang Y, McLeod A. Do health information technology investments impact hospital financial performance and productivity? *International Journal of Accounting Information Systems*. 2018;28:1–13.	2018	US
**58**	**58**	**Lim et al. 2018**	Lim JS, Lim KS, Heinrichs JH, et al. The role of hospital service quality in developing the satisfaction of the patients and hospital performance. *Management Science Letters.* 2018;8(12):1353–1362.	2018	US
**59**	**94**	**Karim et al. 2018**	Karim SA, Pink GH, Reiter KL, Holmes GM, Jones CB, Woodard EK. The Effect of the Magnet Recognition Signal on Hospital Financial Performance. *Journal of Healthcare Management / American College of Healthcare Executives.* 2018;63(6):e131–e146.	2018	US
**60**	**36**	**Glover 2019**	Glover G. Relationships Between Nursing Resources, Uncompensated Care, Hospital Profitability, and Quality of Care.	2019	PhD dissertation
**61**	**78**	**Nagendran et al. 2019**	Nagendran M, Kiew G, Raine R, Atun R, Maruthappu M. Financial performance of English NHS trusts and variation in clinical outcomes: a longitudinal observational study. *BMJ Open*. 2019;9(1),:e021854.	2019	UK
**62**	**40**	**Asagbra et al. 2019**	Asagbra, O. E., Zengul, F. D., & Burke, D. (2019 Patient Engagement Functionalities in U.S. Hospitals: Is Early Adoption Associated with Financial Performance?). Journal of Healthcare Management, 64(6), 381–396.	2019	US
**63**	**70**	**Zhao et al. 2019**	Zhao M, Hamadi H, Rob Haley D, White-Williams C, Liu X, Spaulding A. The Relationship between Health Information Technology Laboratory Tracking Systems and Hospital Financial Performance and Quality. *Hospital Topics.* 2019;97(3):99–106.	2019	US
**64**	**85**	**Akinleye et al. 2019**	Akinleye DD, McNutt LA, Lazariu V, McLaughlin CC. Correlation between hospital finances and quality and safety of patient care. *PLoS One.* 2019;14(8):e0219124. Published 2019 Aug 16. doi:10.1371/journal.pone.0219124	2019	US
**65**	**41**	**Beauvais et al. 2019**	Beauvais B, Richter JP, Kim FS. Doing well by doing good: Evaluating the influence of patient safety performance on hospital financial outcomes. *Health Care Management Review*. 2019;44(1):2–9.	2019	US
**66**	**67**	**Upadhyay et al 2019**	Upadhyay S, Stephenson AL, Smith DG. Readmission Rates and Their Impact on Hospital Financial Performance: A Study of Washington Hospitals. Inquiry: *A Journal of Medical Care Organization, Provision and Financing.* 2019;56:46958019860386. doi.org/10.1177/0046958019860386<br>To include – process measure readmission rate was used.	2019	US
**67**	**30**	**Chakraborty 2020**	Chakraborty S. Healthcare quality and hospital financial performance: A multilevel framework. *Operations and Supply Chain Management.* 2020;13(3):233–243.	2020	theoretical, conceptual paper
**68**	**91**	**Dauser et al. 2021**	Dauser B, Hartig N, Vedadinejad M, Kirchner E, Trummer F, Herbst F. Robotic-assisted repair of complex ventral hernia: can it pay off? *Journal of Robotic Surgery. 2021;*15(1):45–52. doi.org/10.1007/s11701-020-01078-3	2021	Austria
**69**	**42**	**Brooks et al. 2021**	Brooks M, Beauvais BM, Kruse CS, et al. Accreditation and Certification: Do They Improve Hospital Financial and Quality Performance? Healthcare (Basel, Switzerland). 2021;9(7).	2021	US
