# Supplementary material for: The Association Between Hospital Financial Performance and the Quality of Care – A Scoping Literature Review
Source: Int J Health Policy Manag. 2022 Aug 16;11(12):2816–28. doi: 10.34172/ijhpm.2022.6957 (PMC10105205; doi:10.34172/ijhpm.2022.6957)
Supplement: Supplementary file 5 — Overview of the Non-empirical Studies. [file ijhpm-11-2816-s005.pdf]

**Article title:** The Association Between Hospital Financial Performance and the Quality of Care – A Scoping Literature Review

**Journal name:** International Journal of Health Policy and Management (IJHPM)

**Authors' information**: Katarzyna Dubas-Jakóbczyk[1], Ewa Kocot[1], Marzena Tambor[1], Przemysław Szetela[1], Olga Kostrzewska[2], Richard B. Siegrist Jr[3], Wilm Quentin[4,5]*

[1]Health Economics and Social Security Department, Institute of Public Health, Faculty of Health Sciences, Jagiellonian University Medical College, Krakow, Poland.

[2]Institute of Public Health, Faculty of Health Sciences, Jagiellonian University Medical College, Krakow, Poland.

[3]Harvard T.H. Chan School of Public Health, Boston, MA, USA.

[4]Department of Health Care Management, Technische Universität Berlin, Berlin, Germany.

[5]European Observatory on Health Systems and Policies, WHO European Centre for Health Policy Eurostation (Office 07C020), Brussels, Belgium.

(*Corresponding author: Email: Katarzyna.Dubas@uj.edu.pl)

**Supplementary file 5.** Overview of the Non-empirical Studies

## Non-empirical studies extraction table:

| No | Reference number | First author/s and publication year | Type of study | Objective | Results | Conclusions |
|---|---|---|---|---|---|---|
| 1 | **30** | **Chakraborty 2020** | theoretical, conceptual paper | To offer a multi-level conceptual framework that can be used by U.S. hospitals to improve the quality of care and improve the hospital financial performance over the long run period. | The Donabedian's structure - process - outcome paradigm was extended to include different variables that spread across three levels of measurement (micro, meso and macro) and cover long-term effects. Healthcare team cohesion, healthcare team effectiveness and medical treatment expenses were considered as the **structure** (because these variables are concerned with the context of care); the quality of patient care as the **process** (because it denotes the transactions between patients and healthcare providers during the medical treatment and even beyond); and the three variables—patient satisfaction, hospital | Hospital reputation is positively associated with hospital financial performance. Hospitals of repute put significant collective effort and resources to maintain high patient care quality standards in order to keep their high public rankings and patient ratings. The presented conceptual framework can guide multi-level empirical healthcare research on hospital quality. |

| | | | | | | |
|---|---|---|---|---|---|---|
| | | | | | reputation, and hospital financial performance as the **outcomes** (because they refer to the effects of healthcare on the health status of patients and populations). | |
| 2 | **31** | **Fleming & Boles 1994** | theoretical, conceptual paper | To provide and develop a conceptual model of hospital financial and clinical integrity. | The provided model allows to explore the endogeneity of hospital financial and clinical integrity, where the latter includes structure-process-outcomes quality measures. The model also includes organizational and environmental factors. | The model provided an conceptual framework to be tested empirically. The model presents the information on how various clinical integrity aspects (quality of care) affect the financial integrity. |
| 3 | **32** | **Holt et al. 2011** | literature review | Assessment of the impact of organizational factors on the hospital financial performance based on a review of empirical research. | On the basis of 3 analyzed studies, it can be concluded that hospitals that undertook extensive initiatives to improve the quality of services achieved better financial results. Furthermore, "higher quality hospitals have a greater market share and / or lower costs due to greater productivity and efficiency as measured by faster recovery of patients". | Out of the 47 studies included in the review, 3 concerned the relationship between the quality of care and the situation and financial performance of the hospital. Researchers found that there is a strong relationship between patients' perceptions of service quality and financial outcomes. |
| 4 | **33** | **Zengul et al. 2014** | literature review | To investigate the relationship between US hospital financial performance and technologies used in medical services based on a literature review. | Of the 26 studies analyzed, 8 concerned the relationship between the technology used (quality) and financial performance. The 8 analyzed studies provide mixed results:<br>- some studies showed a positive relationship between the technology indicator and the financial performance<br>- long-term studies did not show a significant relationship between the financial performance of hospitals and the technology used. | Based on this review, it is not possible to indicate either a positive or a negative relationship between the technology used and the financial performance of the hospital. Various factors could have contributed to such ambiguous results, one of which is the degree of technology diversification in hospitals. |
| 5 | **15** | **Barnes et al. 2017** | literature review | Investigate the relationship between the quality of hospital care and the financial performance of US hospitals through a systematic literature review. | Based on the included studies, it was found that:<br>- improvement of financial results is related to the improvement of the financial situation of hospitals,<br>- worse financial situation is associated with a higher probability of an event that is unfavorable for the patient and his safety. | There is still little evidence on the relationship between financial performance and quality of hospital care. Perhaps it results from difficulties related to the analysis itself and collecting data, e.g. the presence of confounding factors. The researchers also suggest that adhering to six key areas related to healthcare quality (patient focus, timeliness, efficiency, equity, patient safety and effectiveness) might improve the financial performance and situation of hospitals. |

| | | | | | | |
|---|---|---|---|---|---|---|
| 6 | **16** | **Beauvais & Wells 2006** | literature review | Analysis of the relationship between the financial situation of health care organizations and the quality of care based on a literature review. | Factors such as fiscal margin, medical expenses and profitability turned out to be particularly important financial factors influencing the financial condition of the facility. Additionally, a negative relationship was observed between the administrative costs incurred by the institution and quality. | Almost all analyzed studies showed a significant, favorable relationship between financial factors and the quality of health care. |
| 7 | **34** | **Oner et al. 2016** | literature review | Analyze and synthesize the relationship between external / internal hospital factors and financial outcomes based on a literature review. | Among the 6 distinguished factors used to assess and measure the financial performance of hospitals, the most frequently used were structural factors, and the most frequent relationship tested with regard to financial performance was the relationship between structural factors and the profitability dimension. Most of the obtained results were statistically insignificant. | An analysis of the 81 studies selected for review showed mixed / insignificant results regarding the relationship between internal and external hospital factors and financial outcomes. Perhaps this is due to the differences in , among others, the number and research samples, measurement periods and the presence of various factors in different countries. |
| 8 | **35** | **Cusack 2012** | PhD dissertation | Analysis of the relationship between quality and acute care hospital profitability (relation between quality measures and financial outcomes). | The results showed a positive relationship between financial measure – patient care operating margins – and the two quality indicators i.e. acute myocardial infraction and surgical procedure. Others examined quality measures did not show a significant relationship to the financial indicators or showed mixed effects. Two different analytical methodologies/models have been used: Mixed Linear Model and Generalized Estimating Equation. The results in both models were similar. However, the two models differed when the relationship between the quality indicators and net patient revenues has been examined. | The hospitals with higher scores for the following quality indicators: acute myocardial infraction and surgical procedure had better financial situation. The quality of care affects hospital revenues. However, only acute care hospitals in New Jersey have been analysed. Th higher revenues are connected with a higher quality. The nurse staffing levels have a positive impact on quality – the nurse staffing levels as quality measure showed that higher levels of staffing do not decrease profit margins. |
| 9 | **36** | **Glover 2019** | PhD dissertation | Analysis of the relationship between nursing resources, uncompensated care, hospital profitability and quality of care (overall patient | The following measures have been used: (1) nursing salaries per patient day; (2) cost of uncompensated care as a percentage of net patient revenue; (3) percentage of net income from patient services. The overall patient satisfaction with quality of care received has been used as quality measure. The results of multiple linear regression analysis are as follows: nursing salaries per patient day and percentage of net income from patient services had significant positive effect on the overall patients | Data analysis from 166 acute urban hospitals showed that the hospital resources (including financial) have impact on the quality of patient care (overall patient satisfaction with quality of care). Hospitals that generate a positive net income are able to provide care that increases patient satisfaction. |

| | | | | | | |
|---|---|---|---|---|---|---|
| | | | | satisfaction with quality of care received). | satisfaction with quality of care. The cost of uncompensated care as a percentage of net patient revenue showed a significant negative relationship with the overall patient satisfaction with quality of care. | |
