# Supplementary material for: The Association Between Hospital Financial Performance and the Quality of Care – A Scoping Literature Review
Source: Int J Health Policy Manag. 2022 Aug 16;11(12):2816–28. doi: 10.34172/ijhpm.2022.6957 (PMC10105205; doi:10.34172/ijhpm.2022.6957)
Supplement: Supplementary file 6 — Overview of the Theoretical Frameworks Used in Empirical Studies. [file ijhpm-11-2816-s006.pdf]

**Article title:** The Association Between Hospital Financial Performance and the Quality of Care – A Scoping Literature Review

**Journal name:** International Journal of Health Policy and Management (IJHPM)

**Authors' information**: Katarzyna Dubas-Jakóbczyk[1], Ewa Kocot[1], Marzena Tambor[1], Przemysław Szetela[1], Olga Kostrzewska[2], Richard B. Siegrist Jr[3], Wilm Quentin[4,5]*

[1]Health Economics and Social Security Department, Institute of Public Health, Faculty of Health Sciences, Jagiellonian University Medical College, Krakow, Poland.

[2]Institute of Public Health, Faculty of Health Sciences, Jagiellonian University Medical College, Krakow, Poland.

[3]Harvard T.H. Chan School of Public Health, Boston, MA, USA.

[4]Department of Health Care Management, Technische Universität Berlin, Berlin, Germany.

[5]European Observatory on Health Systems and Policies, WHO European Centre for Health Policy Eurostation (Office 07C020), Brussels, Belgium.

(*Corresponding author: Email: Katarzyna.Dubas@uj.edu.pl)

**Supplementary file 6.** Overview of the Theoretical Frameworks Used in Empirical Studies

## Theoretical or conceptual frameworks applied in empirical research – overview:

| No | Reference number | First author/s and publication year | Theoretical/conceptual framework | Reference/Visualization |
|---|---|---|---|---|
| 1. | **49** | Harkey & Vraciu 1992 | a theoretical model on the impact of quality on profitability via two routes: reduced costs and market gains | 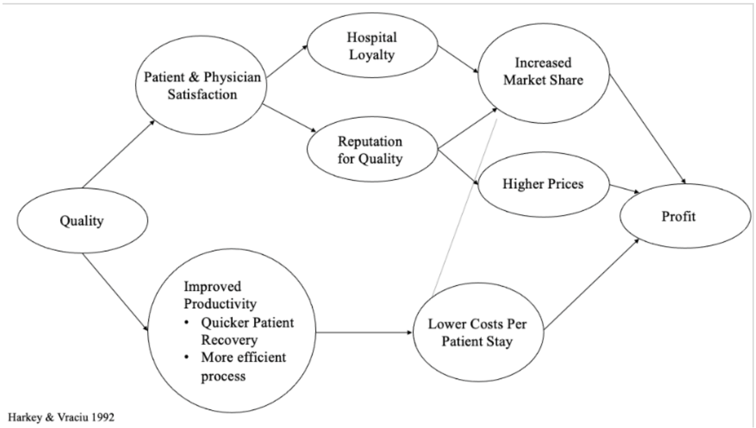 |
| 2. | **55** | Irwin et al. 1998 | **resource-based theory** (Barney 1991) | • Barney, J., 1991. Firm resources and sustained competitive advantage. J. Manage. 17, 99–120. |
| 3. | **57** | Li & Collier 2000 | a theoretical model of relationship between 5 domains: clinical technology, information technology, clinical quality, process quality, and financial performance | |

| | | | | |
|---|---|---|---|---|
| | | | | 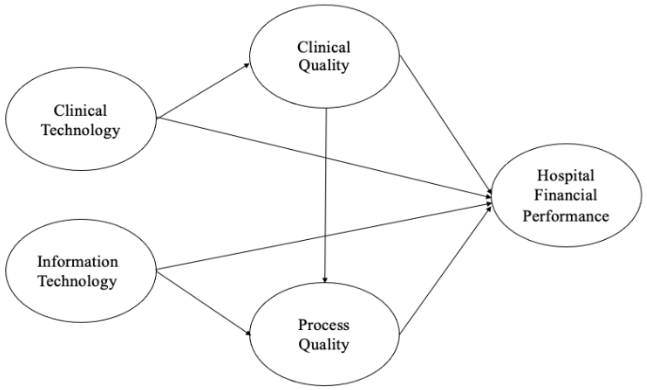<br>Li & Collier 2000 |
| 4. | **72** | Encinosa & Bernard 2005 | a conceptual framework of gradual influence of financial performance on patient safety | |
| 5. | **37** | Alexander et al. 2006 | **theory on control- vs learning-oriented approach to quality** improvement (Sitkin et al. 1994) | • Sitkin, S. B., Sutcliffe, K. M., & Schroeder, R. G. (1994). Distinguishing control from learning in total quality management: A contingency perspective. The Academy of Management Review, 19, 537–564. |
| 6. | **77** | Menachemi et al. 2006 | the **resource-based theory** (Barney 1991): organizations compete based on unique organizational resources that are valuable, rare, difficult to imitate, and nonsubstitutable by other resources | • Barney, I. 1991. "Firm Resources and Sustained Competitive Advantage." Journal of Management 17 (1): 99-120. |
| 7. | **82** | Bazzoli et al. 2007 | **an economic theory of hospital behaviour** (Hoerger 1991; Newhouse 1970) | • Hoerger, T. J. 1991. “Profit” variability in for-profit and not-for-profit hospitals. *Journal of Health Economics* 10(October): 259-89.<br>• Newhouse, J. P. 1970. Towards a theory of nonprofit institutions.<br>• An Economic Model of a Hospital *American Economic Review* 60(1): 64-74. |
| 8. | **74** | Kazley & Ozcan 2007 | **resource dependence theory** (Pfeffer & Salancik 1978): hospitals use EMR for securing patient demand and financial reimbursement | • Pfeffer, J., and C. Salancik. 1978. The Exter-nal Control of Organizations: A Resource Dependence Perspective. New York: Harper&Row. |

| | | | | |
|---|---|---|---|---|
| 9. | **86** | Bazzoli et al. 2008 | **an economic model of a hospital behaviour** (Newhouse 1970) | 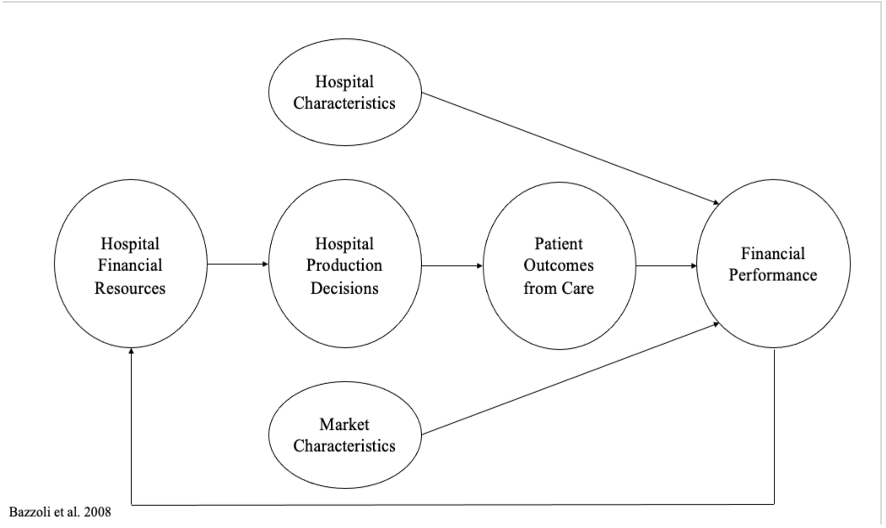<br><br>• Newhouse, J. P. 1970. Towards a theory of nonprofit institutions. An Economic Model of a Hospital *American Economic Review* 60(1): 64-74. |
| 10. | **81** | Zhao et al. 2008 | **an economic theory of hospital behaviour** (Hoerger 1991) on hospitals profits maximalization / surplus targeting involving staff costs constrains | • Hoerger, T. J. 1991. ‘‘Profit’’ Variability in For-Profit and Not-for-Profit Hospitals. Journal of Health Economics10(3):259–289. |
| 11. | **59** | Maiga & Jacobs 2009 | A theoretical framework linking the relationships between 6 dimensions: leadership, clinical quality, process quality, patient satisfaction, cost Improvement, hospital profitability | |

| | | | | |
|---|---|---|---|---|
| | | | | 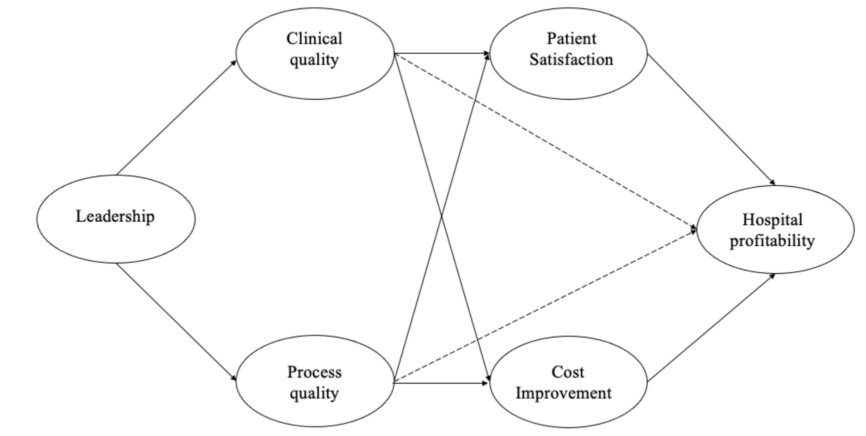<br><br>Maiga & Jacobs 2009 |
| 12. | **73** | Ginn et al. 2011 | **institutional theory** (Meyer and Rowan 1977; DiMaggio and Powell 1983): pressure from healthcare policymakers forces hospitals to adopt EHRs to preserve their legitimacy; **resource dependency theory** (Pfeffer & Salancik 1978): hospitals would adopt EHRs to ensure patient demand | • DiMaggio, P. I., and W. W. Powell. 1983. "TheIron Cage Revisited: Institutional Iso-morphism and Collective Rationality in Organizational Fields." American Sociological Review A8 i^2): 147-60.<br>• Meyer, J. W., and B. Rowan. 1977. "Institution-alized Organizations: Formal Structure as Myth and Ceremony." American Journal of Sociology 83 (2): 340-63.<br>• Pfeffer, J., and C. Salancik. 1978. The Exter-nal Control of Organizations: A Resource Dependence Perspective. New York: Harper&Row. |
| 13. | **79** | Navathe et al. 2012 | **an economic model of a hospital behaviour** (Newhouse 1970): decreasing reimbursement reduces providers' incentives to incur the costs necessary to provide levels of quality above the profit-maximizing level and beyond the minimum level implied by professional standards, regulations, etc. | • Newhouse J. P. (1989). Do unprofitable patients face access problems? Health Care Financing Review, 11, 33-42. |
| 14. | **64** | Reiter et al. 2012 | economic models of production: hospitals marginal revenues are equal to their marginal costs (Avery & Shultz 2007). | • Avery, G., and J. Shultz. 2007."Regulation, Financial Incentives, and the Production of Quality." American Journal of Medical Quality22 (4): 265–73. |

| | | | | |
|---|---|---|---|---|
| 15. | **47** | Everhart et al. 2013 | **resource-based theory** (Barney 1991) | • Barney J. Firm resources and sustained competitive advantage. Journal of Management. 1991; 17(1):99–120. |
| 16. | **75** | Lindrooth et al. 2013 | **a model on hospitals cross-subsidizing quality in unprofitable services** using the financial surplus from other services depending on the financial surplus availability (David et al. 2011). | • David, G., R. C. Lindrooth, L. A. Helchem, and L. R. Burns. 2011.“Do Hospitals Cross Subsidize?” NBER Working Papers 17300. |
| 17. | **84** | Turner et al. 2015 | a conceptual relationship between VBP adjustment and financial performance | 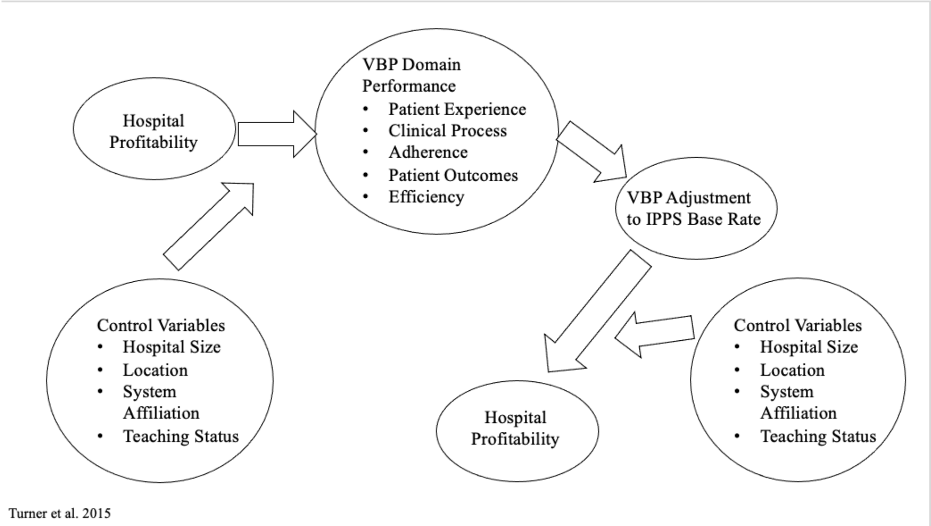 |
| 18. | **88** | Collum et al. 2016 | **corporate financial theory** (Copeland et al 2005). | • Copeland, T.E., Weston, J.F., &Shastri, K. (2005). Financial the oryand corporate policy (4th ed.). Reading, MA: Addison-Wesley. |
| 19. | **90** | Crowe et al. 2017 | a leadership development systems perspective using the **High-Performance Work Practices (HPWP) theory** (Huselid 1995) that is an extension of the original **resource-based theory (**Barney 1991) of the firm. | • Huselid M. The impact of human resource management practices on turnover, productivity, and corporate financial performance. Acad Manag J1995; 38: 635–872.<br>• Barney J. Firm resources and sustained competitive advantage. Journal of Management. 1991; 17(1):99–120. |

| | | | | |
|---|---|---|---|---|
| 20. | **65** | Richter & Muhlestein 2017 | the **service-profit chain framewor**k (Heskett et al. 1994), linking the customers satisfaction/loyalty with revenues growth. | • Heskett, J., Jones, T. O., Loveman, G. W., & Schlesinger, L. A. (1994). Putting the service-profit chain to work. Harvard Business Review,72(2), 164Y174. |
| 21. | **94** | Karim et al. 2018 | A theoretical framework - **signaling theory -** signalling aims to reduce the imbalance of information between two parties (also known as information asymmetry) (Erdem 1998). If the signal is effective, an overall improvement in hospital financial performance is expected. | • Erdem, T. T. (1998). Brand equity as a signaling phenomenon. Journal of Consumer Psychology, 7(2), 131–157. |
| 22. | **58** | Lim et al. 2018 | a conceptual model of the relationships between hospital service quality, patient satisfaction, hospital utilization, and hospital financial performance | 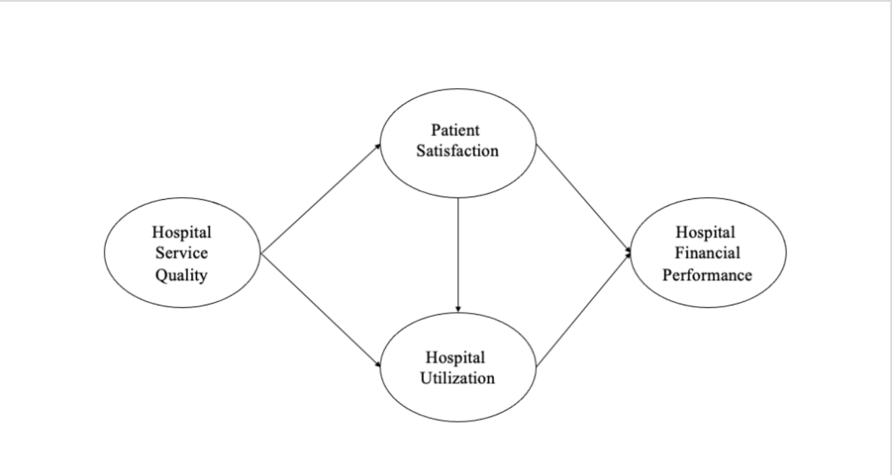<br>Lim et al. 2018 |

| | | | | |
|---|---|---|---|---|
| 23. | **68** | Wang et al. 2018 | theoretical model on the relationships between IT investments, financial performance, and intermediate business processes, i.e. electronic health records adoption | 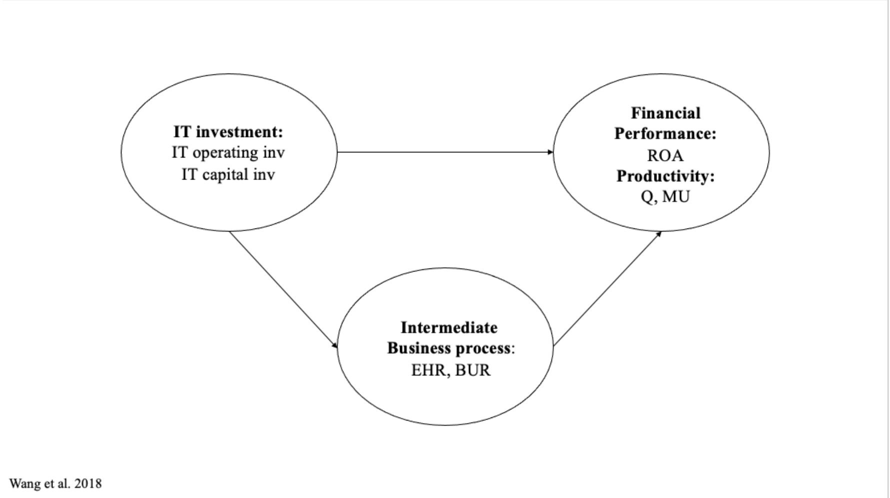 |
| 24. | **85** | Akinleye et al. 2019 | Rogers' (2003) work on **diffusion of innovations** (HIT adopters' groups) | |
| 25. | **40** | Asagbra et al. 2019 | a conceptual framework on combining multiple measures of both FP and Q into predictive model | • Rogers, E. M. (2003). Diffusion of innovations. New York, NY: Free Press; 551. |
| 26. | **41** | Beauvais et al. 2019 | a theoretical model on the connection between service quality improvement, cost reductions, and profitability (Rust et al 1995) | • Rust, R. T., Zahorik, A. J., & Keiningham, T. L. (1995). Return on quality (ROQ): Making service quality financially account able. The Journal of Marketing,58Y70. |
| 27. | **67** | Upadhyay et al 2019 | **Resource dependence theory (**Pfeffer & Salancik 1978): organization's survival relies on its ability to acquire resources from the external environment. When resources become less available, organization adapt strategies to survive, i.e., readmission reduction. | • Pfeffer, J., and C. Salancik. 1978. The Exter-nal Control of Organizations: A Resource Dependence Perspective. New York: Harper&Row. |
| 28. | **81** | Zhao et al. 2019 | Donabedian's quality framework (1988, 2003) | • Donabedian, A. The quality of care. How can it be assessed? 1988. Arch. Pathol. Lab. Med. 121 (11): 1145–1150. |

| | | | | |
|---|---|---|---|---|
| | | | | • Donabedian, A. 2003. An introduction to quality assurance in health care. New York, NY: Oxford University Press |
| 29. | **42** | Brooks et al. 2021 | **institutional isomorphism based on institutional theory** – process whereby organizations exposed to the same institutional environments tend to adopt similar characteristics and practice | • DiMaggio, P.J.; Powell, W.W. The iron cage revisited: Institutional isomorphism and collective rationality in organizational fields. Am. Sociol. Rev. 1983, 48, 147–160. |
